# *Nxf1* Natural Variant E610G Is a Semi-dominant Suppressor of IAP-Induced RNA Processing Defects

**DOI:** 10.1371/journal.pgen.1005123

**Published:** 2015-04-02

**Authors:** Dorothy Concepcion, Kevin D. Ross, Kasey R. Hutt, Gene W. Yeo, Bruce A. Hamilton

**Affiliations:** 1 Department of Cellular and Molecular Medicine, Moores UCSD Cancer Center and Institute for Genomic Medicine, University of California, San Diego School of Medicine, La Jolla, California, United States of America; 2 Department of Medicine, University of California, San Diego School of Medicine, La Jolla, California, United States of America; 3 Biomedical Sciences Graduate Program, University of California, San Diego School of Medicine, La Jolla, California, United States of America; 4 Stem Cell Program, University of California, San Diego School of Medicine, La Jolla, California, United States of America; Stanford University School of Medicine, UNITED STATES

## Abstract

Endogenous retroviruses and retrotransposons contribute functional genetic variation in animal genomes. In mice, Intracisternal A Particles (IAPs) are a frequent source of both new mutations and polymorphism across laboratory strains. Intronic IAPs can induce alternative RNA processing choices, including alternative splicing. We previously showed IAP I∆1 subfamily insertional mutations are suppressed by a wild-derived allele of the major mRNA export factor, *Nxf1*. Here we show that a wider diversity of IAP insertions present in the mouse reference sequence induce insertion-dependent alternative processing that is suppressed by *Nxf1^CAST^* alleles. These insertions typically show more modest gene expression changes than de novo mutations, suggesting selection or attenuation. Genome-wide splicing-sensitive microarrays and gene-focused assays confirm specificity of *Nxf1* genetic modifier activity for IAP insertion alleles. Strikingly, CRISPR/Cas9-mediated genome editing demonstrates that a single amino acid substitution in Nxf1, E610G, is sufficient to recreate a quantitative genetic modifier in a co-isogenic background.

## Author Summary

Transposable elements, including endogenous retroviruses, have long been hypothesized as a substrate for creating or modulating gene regulatory networks, particularly through effects on transcription. However, several classes of elements are also known to affect alternative RNA processing events. We previously showed that the major allele of nuclear export factor *Nxf1* in *Mus musculus castaneus* mice acts as a semi-dominant suppressor of de novo mutations caused by intracisternal A particle (IAP) endogenous retroviruses that integrate into introns, disrupting normal RNA processing. Here we show that this suppressor allele of *Nxf1* can coordinately modify gene expression phenotypes at several endogenous loci in the C57BL/6 mouse reference genome that contain IAP sequences in their introns. This quadruples the number of known insertional events modified by *Nxf1* and extends the effect beyond overt mutations. We previously used transgenic mice and viral vector mediated overexpression to demonstrate *Nxf1* as the modifier gene for de novo insertions. Here we use direct genome editing in mouse one cell embryos to create custom germline alleles at the endogenous *Nxf1* locus to show that a specific amino acid substitution, E610G, quantitatively accounts for the *Nxf1* modifier gene activity.

## Introduction

Endogenized retroviruses and other molecular parasites frequently influence expression of host genes at sites of insertion. Chromosomal insertions of these mobile elements can alter initiation, splicing, or termination of host gene transcripts, in quality or amount. Remnants of ancient insertion and transposition events that survived selection are thought to have shaped gene expression patterns in modern animals substantially [1–3]. In populations where mobile elements remain highly active, such events can account for a substantial fraction of functional polymorphism and spontaneous mutations. Two families of elements in laboratory mice, Intracisternal A Particle (IAP) and MusD/Early Transposon (ETn) families account for 10–20% of spontaneous mutations [4–6], depending on strain background [6]. While some of these mutations interrupt coding exons [7] or induce novel patterns of transcription [8–11], the majority comprise intronic insertions that introduce alternative splicing or transcript termination (or both), resulting in quantitative loss of normal host gene products.

Alternative processing of nascent transcripts is regulated at several levels [12]. Pre-mRNA splicing typically occurs co-transcriptionally, regulated by a variety of DNA and RNA binding factors that together defines and acts on constitutive exons. Transcriptional initiation complexes may assemble splicing factors on the Pol II complex, resulting in promoter-dependent alternative splicing [13–15]. Elongation rate of the RNA polymerase complex may influence alternative splicing by regulating the appearance of downstream acceptor sites relative to the splicing kinetics for weaker upstream sites [16, 17]. In addition, recent single-molecule imaging data supports post-transcriptional splicing for at least some alternative splice sites [18]. Identification of post-transcriptional alternative splicing events suggests opportunities for regulation by nuclear ribonucleoprotein (RNP)-associated proteins that are not normally found in the nuclear speckles associated with constitutive splicing.

We previously reported a wild-derived variant of the mouse mRNA nuclear export factor gene *Nxf1* within the *Modifier-of-vibrator-1* (*Mvb1*) locus as a genetic modifier for each of six mutations caused by insertion of an IAP element into an intron of a host gene [19, 20]. The suppressing allele, *Nxf1* ^*CAST*^, defines the major haplotype in wild isolates of *Mus musculus castaneus*. For each of the six mutations, genetic suppression altered the balance of alternative RNA processing between virus-dependent isoforms and splicing to the 3’ exon. This mechanism appeared highly selective, operating on sense-strand IAPs (six out of seven tested), but not MusD/ETn (0/6), MuLV (0/1), VL30 (0/1), or L1 LINE (0/1) insertions. These studies also identified an exception to the rule: *Atrn* ^*mgL*^, which includes a “full-length” IAP, was not suppressed, in contrast to the more frequent IΔ1 class [21] in the six suppressed mutations [19]. We proposed that sequences deleted in IΔ1 elements might in full-length elements mediate additional repressive events that are epistatic to the apparent splicing defects modified by *Nxf1* ^*CAST*^, but acknowledged that local genomic context could also play a role. We also proposed that *Nxf1* ^*CAST*^ effects on alternative splicing are highly specific, but genome-wide analysis of *Nxf1* ^*CAST*^ effects on either general alternative splicing or alternative splicing at genes whose reference allele includes an IAP element have not been reported.

Here we show that *Nxf1* ^*CAST*^ has modifier gene activity toward IAP insertion alleles present in the C57BL/6J (B6) reference genome–this includes several full-length IAPs, an IAP in *Adamts13* with a novel deletion but otherwise high sequence similarity to the non-suppressed element in *Atrn* ^*mgL*^, and two other IAP deletion classes outside of the IΔ1 group. Quantification of well-characterized alternative splicing events, including cassette exon, retained intron, alternative splice donor, and alternative splice acceptor sites detect no *Nxf1* modifier effect at non-IAP introns, further supporting the specificity of *Nxf1* ^*CAST*^ activity toward IAP insertion alleles. We also demonstrate by genome editing that a single nucleotide substitution, encoding a glutamate to glycine amino acid replacement in the carboxyterminal UBA-like domain, is sufficient to explain the semi-dominant modifier phenotype of *Nxf1* ^*CAST*^. These results expand the range of IAP family elements modified by *Nxf1* variant alleles and identify a single residue in Nxf1 protein whose effect on stability, kinetics, or interactions explain the modifier mechanism.

## Results

### *Nxf1* ^*CAST*^ modifies *Adamts13* ^*S*^, an atypical deleted-IAP allele in B6 mice

**Fig 1:**
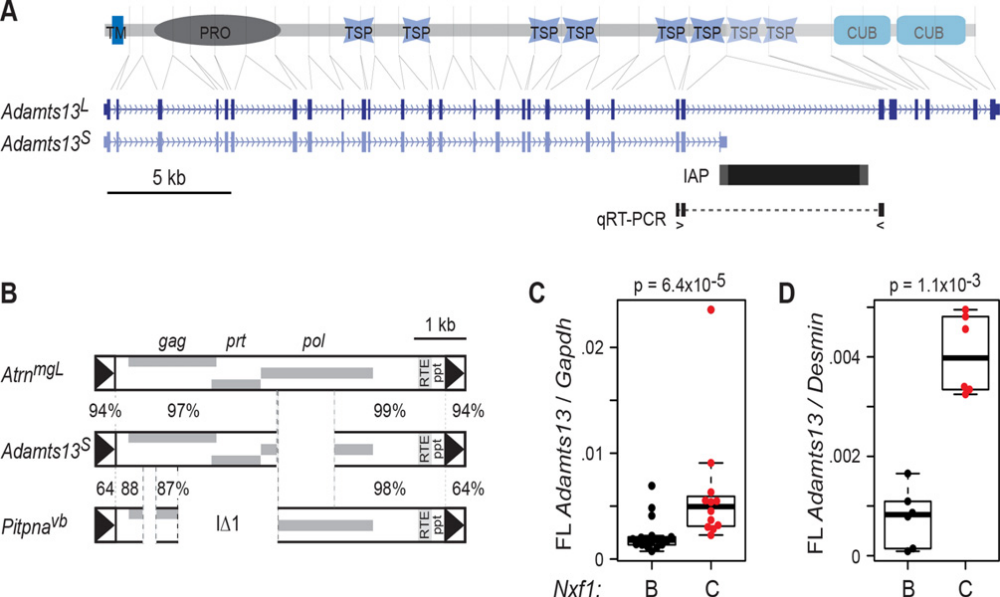
*Adamts13* ^*S*^ is suppressed by congenic *Nxf1* ^*CAST*^. **(A)** Alignment of Adamts13 protein domains to their corresponding exons in *Adamts13* ^*L*^ and *Adamts13* ^*S*^ shows loss of two thrombospondin (TSP) and two CUB domains from *Adamts13* ^*S*^, which terminates in an intronic IAP sequence. TM, transmembrane segment; PRO, protease domain. Locations of primers to detect exon 24 to exon 25 splicing around the IAP are indicated. **(B)** The *Adamts13* ^*S*^ IAP shares greater sequence similarity with the *Atrn* ^*mgL*^ full-length IAP than with IΔ1 elements, an example of which is shown. Percent nucleotide identity is shown for indicated segments. **(C)** Quantitative RT-PCR for exon 24-exon 25 splice junction relative to *Gapdh* in B6 x BALB/c F2 mice homozygous for both *Adamts13* ^*S*^ and the indicated congenic allele of *Nxf1* shows substantial increase in the *Nxf1* ^*CAST*^ congenic allele (p = 6.4x10^-5^, Wilcoxon rank sum test). **(D)** Similar results were obtained for a smaller number of animals from the B6 congenic line (p = 1.1x10^-3^, Wilcoxon rank sum test). Normalization to *Desmin* as a cell type-specific marker increased the separation of values. Each dot in the box plots represents the mean of technical replicates for one biological sample.

Because congenic *Nxf1* ^*CAST*^ showed modifier activity against several insertions of the IAP IΔ1 subfamily, but not against a single example of a full-length element [19, 20], we tested its activity on a well-studied IAP allele of a different class (Fig. 1). *Adamts13* encodes a large circulating protease responsible for processing multimeric von Willebrand factor (vWF) in vivo [22, 23]; mutations in human *ADAMTS13* cause thrombotic thrombocytopenic purpura [24] and variations in ADAMTS13 activity are associated with other thrombotic abnormalities [25, 26]. In the *Adamts13* ^*S*^ allele present in several inbred strains, an IAP insertion into intron 24 results in an alternatively spliced, stable transcript that terminates in viral sequences, but lacks downstream exons encoding the final two thrombospondin repeats and two CUB (C1r/C1s, Uegf, Bmp1) domains [27]. A very small amount of residual exon 24 to exon 25 splicing can be detected by a quantitative reverse transcription–polymerase chain reaction (qRT-PCR) assays (Ref. 27 and Fig. 1A). The *Adamts13* ^*S*^ IAP sequence is highly similar to the *Atrn* ^*mgL*^ IAP (Fig. 1B), but differs in having a 1131-bp deletion in the *pol* coding region (Fig. 1B), distinct from and smaller than the classical IΔ2 deletion [21]. Of 2329 bp among 107 aligned sites that distinguish the *Atrn* ^*mgL*^ IAP from the IΔ1 elements, only 33 sites (61 bp) are not shared between *Atrn* ^*mgL*^ and *Adamts13* ^*S*^ IAPs.

Congenic *Nxf1* ^*CAST*^ alleles increased the amount of *Adamts13* RNA that is correctly spliced from exon 24 to exon 25. Since *Adamts13* is most robustly expressed by hepatic stellate cells [28, 29], we assayed RNA from replicate sets of liver samples. Measurements in previously collected samples from 29 F2 progeny of a BALB/c X C57BL/6J–*Nxf1* ^*B6*^/*Nxf1* ^*CAST*^ intercross [19], in which the *Adamts13* ^*S*^ allele was fixed, showed a significant increase in full-length *Adamts13* relative to standard reference genes (*Gapdh* or *Ppia*) in animals with the *Nxf1* ^*CAST*^ allele, although variance in the measurement was unusually high (Fig. 1C). To clarify the effect of *Nxf1* ^*CAST*^, we examined qRT-PCR from liver RNA of C57BL/6J–*Nxf1* ^*CAST*^ congenic stock, using *Desmin* as a cell type-specific reference gene to control for proportion of hepatic stellate cells. This confirmed an increase in fully spliced *Adamts13* in *Nxf1* ^*CAST*^ animals, with much lower variance (Fig. 1D). The two experiments together indicate ~2-4-fold elevation in abundance of full-length *Adamts13* in *Nxf1* ^*CAST*^ compared with *Nxf1* ^*B6*^.

### *Nxf1* ^*CAST*^ suppresses IAPs that correlate with reduced expression

The C57BL/6J reference genome includes more diverse examples of sense-oriented IAPs in host gene introns. To allow a more comprehensive survey of IAP structural classes that can be modified by *Nxf1* ^*CAST*^, we systematically identified IAP elements using a combination of sequences that are conserved across IAP subfamilies and RepBase annotations (S1 Fig). Comparison of IAP and host gene strand orientation confirms the well-known orientation bias [20, 30] against sense-strand insertions (323 sense vs. 969 antisense) and shows a significant difference in size distribution, with sense-oriented insertions having a larger proportion of elements ≤ 1 kb (210/323, compared with 525/969 antisense; p = 7.3x10^-4^, Fisher’s exact test). The set of sense-oriented insertions was manually curated to remove non-intronic events (mostly short IAP sequences in 3’ ends that were otherwise homologous with 3’ ends in corresponding human or rat genes) and likely annotation errors (mostly tandem gene duplications with the IAP placed between duplicate copies). Of these, 85 were > 3 kb, 58 of which had been previously reported [31] (S2 Fig).

**Fig 2:**
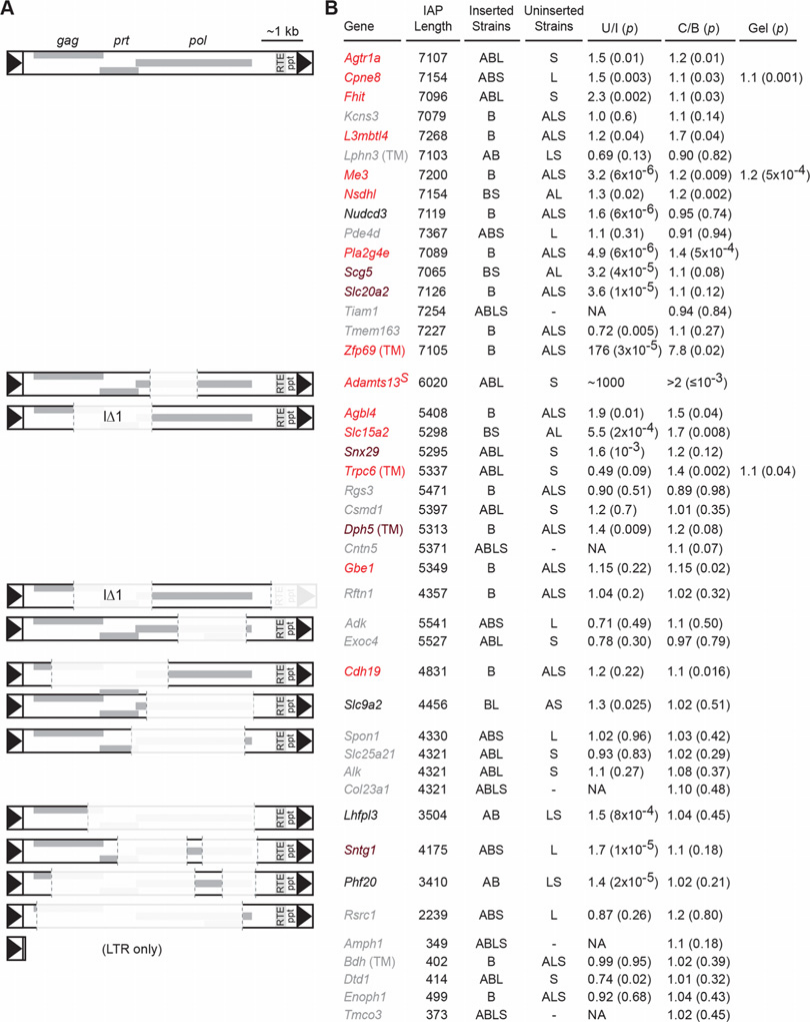
*Nxf1* ^*CAST*^ congenic allele suppresses a diversity of IAP elements in the B6 genome. **(A)** Schematic diagrams illustrate sequence compositions of IAP elements in this study. Deleted segments in each elements compared with full-length consensus are shown at 10% opacity. **(B)** For each structural class, genes whose expression level was tested are indicated. Genes measured with TaqMan assays are indicated by (TM); all others measured by real-time fluorescence stimulation with SYBR green. Gene symbols are color coded: grey for no significant differences between strains that have (Inserted) and strains that lack (Uninserted) each IAP, black for no evidence of suppression by *Nxf1* of an observed strain difference, brown for a non-significant trend toward suppression, and red for significant evidence of suppression by *Nxf1* ^CAST^. Strains that include (Inserted) or lack (Uninserted) each IAP are indicated: A, A/J; B, C57BL/6J (B6); L, BALB/cJ; S, 129S1/SvImJ. Relative expression ratios for each strain are expressed as Uninserted/Inserted (U/I). Relative expression ratios for *Nxf1* congenic animals homozygous for either *Nxf1* ^*CAST*^ (C) or *Nxf1* ^*B6*^ (B) allele are expressed as C/B, such that insertions suppressed by the *Nxf1* ^*CAST*^ allele have values >1. P-values from a two-tailed Wilcoxon-Mann-Whitney test for strain difference (U/I, unpaired samples) or a one-tailed Wilcoxon signed rank test for *Nxf1* ^*CAST*^-dependent suppression (C/B, littermate paired samples) are given in parentheses. Three genes were also confirmed by fluorescence intensity of isoform-specific bands on agarose gels using 3-primer assays designed independently of the qRT-PCR assays.

Intronic IAP elements in the reference genome predict decreased expression relative to strains that lack the insertion (Fig. 2). We designed unique qRT-PCR assays for exon-exon splicing across 49 IAP-containing introns, of which 41 passed initial quality control measures (4 solo LTRs and 36 larger elements). Commercial hydrolysis probe (TaqMan) assays were purchased for 5 additional sites (1 solo LTR and 4 larger elements). In draft assemblies of A/J, BALB/c, and 129S1 genomes available from the Wellcome Trust Sanger Institute [32, 33], 39 insertions >3 kb and all 5 solo LTR insertions were absent in at least one strain. While other factors may influence strain-dependent expression of any given locus, the presence of sense-strand insertions >3 kb correlated with lower levels of the assayed splice junction. Among 39 polymorphic sites, 20 had significantly lower expression in strains with the insertion, 2 had nominally significant differences in the opposite direction, and 17 were not significantly different. Effect sizes of significant difference ranged from 1.2 to 1000-fold in relative expression. These results confirmed that intronic IAPs fixed in the B6 genome correlate with reduced expression of the correctly processed RNA.

Congenic *Nxf1* ^*CAST*^ alleles increased relative expression of processed RNA at these same sites. We assayed correctly spliced expression at 44 sites in paired brain samples homozygous for either *Nxf1* ^*B6*^ or *Nxf1* ^*CAST*^ alleles (Fig. 2). The effect of *Nxf1* genotype on level of correctly spliced RNA was independently significant at 14 sites, including full-length, IΔ1, and other deletion classes of IAPs. Class-specific statistics and false discovery rates are listed in Table 1 and S1 Table. Notably, *Nxf1* ^*CAST*^ also suppressed the effect of an IAP in an AT/AC intron in *Gbe1*, suggesting that suppression is not dependent on the major spliceosome. The magnitude of statistically supported effects ranged from 1.1 (*Cdh19*) to 7.8 (*Zfp69*) in these assays. Furthermore, pooled data from full-length and IΔ1 insertion sites that showed a significant difference between inserted and uninserted strains, but not individually between *Nxf1* alleles, nonetheless showed group-wise significance for suppression by *Nxf1* ^*CAST*^ (p = 0.0012, Wilcoxon signed rank test with continuity correction). These results demonstrate that the *Nxf1* congenic alleles have genetic modifier activity toward a diverse set of IAP elements and genomic contexts.

**Table 1:** Suppression of distinct IAP structural classes by *Nxf1* ^*CAST*^.

IAP class*	Group-wise p**	FDR***
>7 kb	2.1 x 10^-7^	1.1 x 10^-6^
IΔ1	3.4 x 10^-4^	8.6 x 10^-4^
Δpol^1^	0.43	0.50
Solo LTR	0.50	0.50
other	0.0053	0.0088

* Full list of elements by class is given in S1 Table

** p-values from the Wilcoxon rank sum test for all paired measurements in the class

*** False Discovery Rate q-value from the Benjamini-Hochberg method.

^1^ Deletions that remove at least half of the *pol* ORF, but less than half of the adjacent *prt* ORF.

### *Nxf1* genotype does not influence alternative splicing choice at non-IAP sites

**Fig 3:**
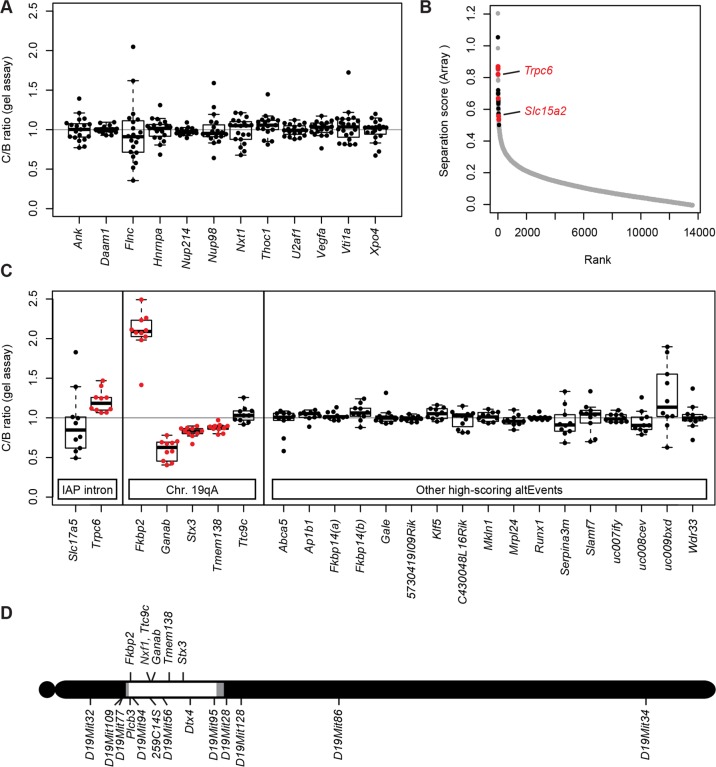
Nxf1 alleles do not affect alternative processing at non-IAP sites. **(A)** The ratio of alternative splice forms for 12 candidate genes was measured by in-gel fluorescence after PCR from congenic *Nxf1* ^*CAST*^ or *Nxf1* ^*B6*^ allele homozygotes. Box-and-whiskers plots show the ratio ratio between paired samples of alternate genotypes (C/B ratio). Ratios of individual sample pairs are plotted as points over each box. **(B)** Three samples of each genotype were hybridized to Affymetrix arrays to interrogate 13,002 alternative RNA events. Absolute value of the separation score (log_2_ of the inclusion/exclusion rate for *Nxf1* C/B paired samples) between alleles is plotted by rank across all probe sets. Events that were confirmed in gene-specific assays on additional samples are indicated in red, events that were tested but not confirmed are in black. Positions of *Trpc6* and *Slc15a2* events tested in Fig. 2 are indicated. **(C)** Ratios between paired samples, as in panel A, for alternative splicing events predicted by the arrays. Significant differences, plotted in red, are either events at IAP-containing introns or in genes within the Nxf1 congenic region on chromosome 19A. **(D)** Diagram of chromosome 19 shows the positions of genes with alternative outcomes in panel (C) relative to the congenic interval homozygous for CAST/EiJ alleles in all *Nxf1* ^*CAST*^ samples (white), intervals between informative markers or heterozygous in some samples (gray), and homozygous for B6 alleles (black).

To test further the specificity of *Nxf1* variant effect on alternative splicing, we examined 12 alternative splicing choices at well-characterized alternative splice sites [34] and alternative splice sites in genes with known functional connections to Nxf1, reasoning that these might be most likely to be influenced by variation in *Nxf1*. None of these introns contain sense-oriented IAP sequences. RT-PCR competition assays that amplify both alternative products were used and the relative ratio of alternative products in each sample quantified by fluorescence imaging after gel electrophoresis (Figs. 3A and S3). No significant differences were observed among 12 candidate sites tested.

As a more general test of modifier activity on non-IAP alternative splicing choices, we measured brain RNA samples from three same-sex littermate pairs from the *Nxf1* congenic stock using genome-wide splicing-sensitive microarrays (Fig. 3B). The Affymetrix MJAY arrays interrogated 13,002 known or imputed alternative junctions in mouse genes, including alternative splice site choices and alternative 5’ and 3’ ends. Approximately 1868 events were called with high confidence (q≤0.05) in this experiment. Using established computational methods [35] to analyze this data, we identified only 43 sites with significant deviations between paired samples (Separation score absolute value >0.5 [~1.4-fold change] and False Discovery Rate q<0.05). Of these, 10 were within the CAST/Ei congenic interval on chromosome 19; due to the high polymorphism rate between B6 and CAST, these are likely due to either mismatch with the probe sequence or to intrinsic allelic differences independent of *Nxf1*. Interestingly, three of the remaining 33 sites included sense-oriented IAP insertions in the corresponding intron: *Trpc6*, *Slc15a2*, and *Slc17a5*, although most IAP-related alternative RNA junctions were not represented in the array.

To test whether the sites implicated by the array analysis were replicable differences rather than probe sequence polymorphisms or statistical fluctuation across a large number of measurements, we designed gel-based RT-PCR assays across each intron for 12 of the most extreme scores on each end of the distribution (Fig. 3C). Of these, four sites tested from chromosome 19 (*Fkbp2*, *Ganab*, *Stx3*, and *Tmem138*), showed significant differences, suggesting an intrinsic processing difference between B6 and CAST/Ei alleles in those genes. Of three IAP-containing introns detected by the arrays *Slc15a2* and *Trpc6* were confirmed by either qRT-PCR (Fig. 2) or both qRT-PCR and the gel assay, while *Slc17a5* could not be confirmed. Thus, 6 out of 8 IAP or chromosome 19 sites identified by the MJAY arrays replicated in an independent assay. By contrast, none of 17 assays for unlinked, non-IAP splice sites showed a nominally significant difference between *Nxf1* alleles. Lack of replication for these events, compared with very high replication rates for IAP-containing events and sites within the *Nxf1*-congenic interval on chromosome 19 (Fig. 3D), further suggest that very few if any non-IAP sites are sensitive to this *Nxf1* allele, consistent with discovery rates seen in previous experiments with this array platform [36–39]. While these data cannot rule out the possibility of a non-IAP alternative processing target for *Nxf1* modifier activity at an unmonitored site or below the limit of detection, together they show that *Nxf1* ^*CAST*^ modifier activity does not act broadly on alternative splicing and provides further evidence for very strong IAP selectivity.

### Construction of *Nxf1* E610G mice by genome editing in mouse one cell embryo

**Fig 4:**
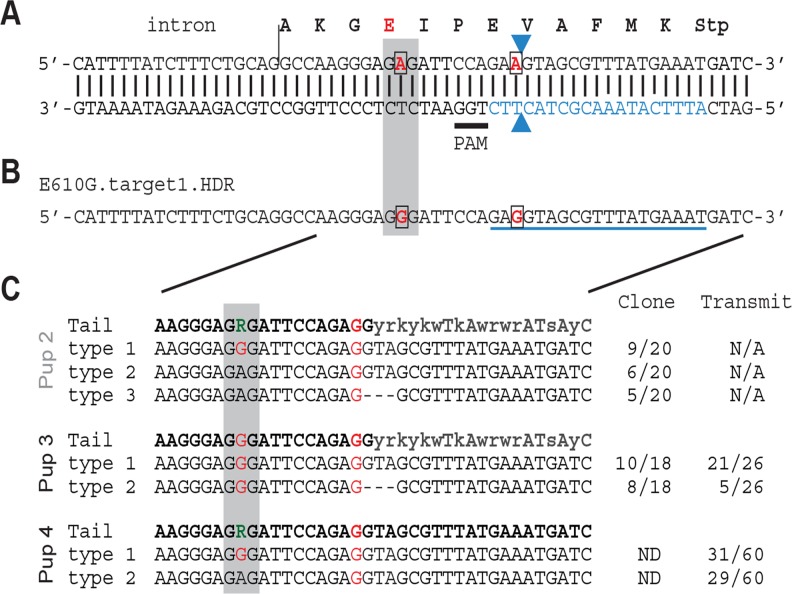
*Nxf1* E610G genome editing in mouse embryos. **(A)** Translation and genomic sequence surrounding E610G targeted for editing. Codon 610 is shaded grey, targeted bases are in red and boxed, guide RNA sequence is in blue, the presumed Cas9 cut site shown by arrowheads, and the PAM sequence is underlined. **(B)** Partial sequence of the repair template oligonucleotide shows the two targeted base changes, including one in the guide RNA sequence. **(C)** Sequences obtained by PCR from tail DNA of one dead (Pup 2) and two live (Pups 3 and 4) G0 pups. Sequence from cloned PCR products (Pups 2 and 3) confirmed an equivalent 3-bp deletion (ΔVal) on one allele in each animal. Sequence from offspring confirmed transmission of each edited allele from the two viable founders.

The precise genetic variant or variants responsible for the modifier activity of *Nxf1* have not been previously determined. As *Nxf1* steady-state RNA and protein levels did not appear different between alleles, we reasoned that one or both of two amino acid substitutions that differentiate the alleles was likely causal. Because the E610G variant occurred in a highly-conserved domain and appeared to be the last variant that arose in a 19-marker haplotype before that haplotype rose to high frequency in wild mice [20], we targeted this site for genome editing in mouse one cell embryos, using a synthetic Cas9 mRNA and a single guide RNA (Fig. 4A) co-injected with an oligonucleotide template for homology-dependent repair (Fig. 4B), essentially as described [40]. Two potential founders survived. Each survivor was male and heterozygous for two distinctly edited alleles–(1) a correctly edited allele containing both the E610G polymorphism and a silent polymorphism in the sgRNA homology to deter further cleavage, and (2) either a “pseudo-edited” allele, carrying the induced silent polymorphism, or the E610G edit together with a 3-bp deletion of the adjacent valine codon (Fig. 4C). Both males bred and transmitted each allele. All four transmitted alleles were detected by PCR sequencing tail-clip DNA from the founders and their offspring. Sequencing of 17 predicted [41] off-target cleavage sites from the same samples identified no additional mutations, suggesting that off-target editing was not frequent in either founder.

### *Nxf1* E610G edited allele recapitulates the *Modifier of vibrator*

**Fig 5:**
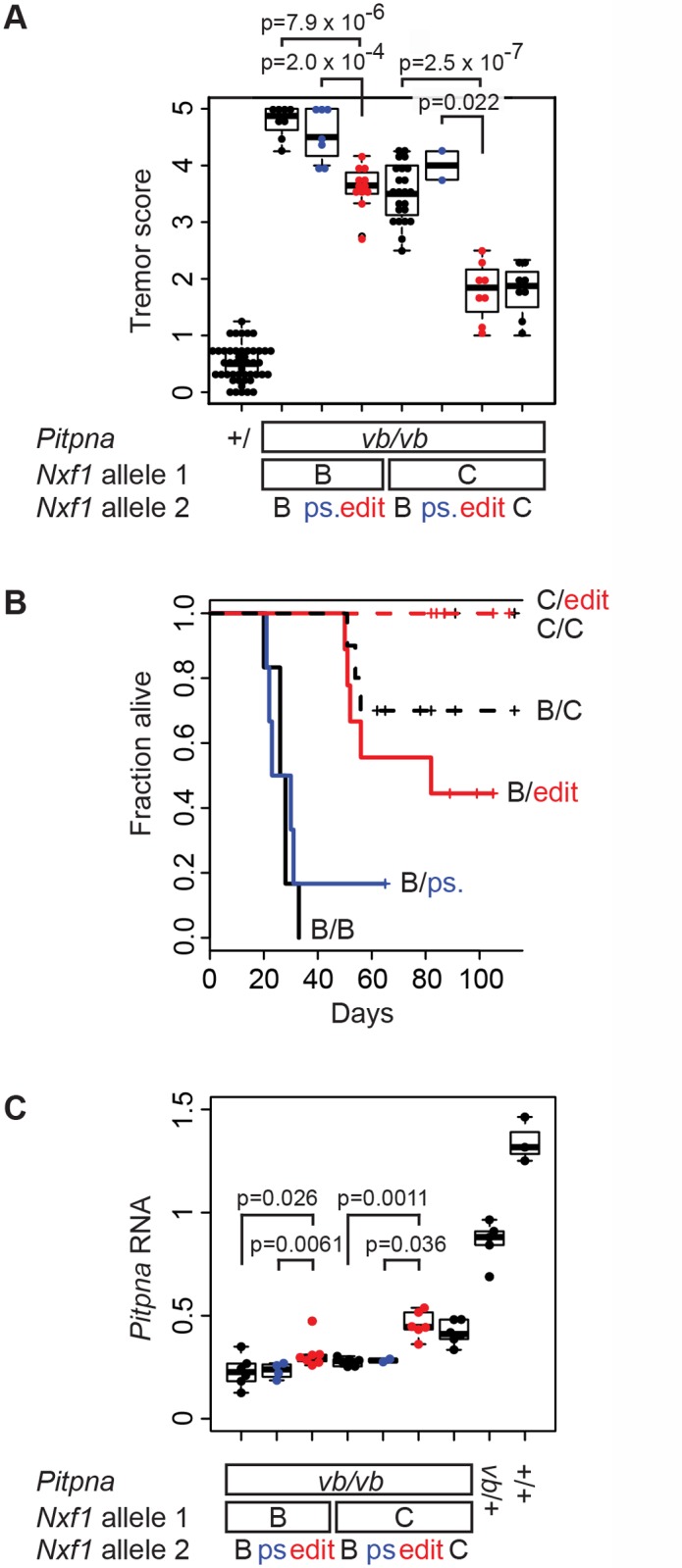
E610G variant accounts for *Modifier-of-vibrator* activity of *Nxf1*. **(A)** The E610G edited allele decreased tremor severity. Tremor scores for *vibrator* mutant and littermate control animals of the indicated genotypes are plotted. Each point represents the mean observer score for one animal. P-values for the single hypothesis tests that one copy of the edited allele significantly reduced tremor relative to the unedited or pseudo-edited (ps.) B6 allele are shown. **(B)** The E610G edited allele increased genotype-dependent survival of *vibrator* animals. Kaplan-Meier plots show fraction alive over a ~3-month observation period. Heterozygotes for edited alleles are drawn in red, pseudo edited in blue. Censored animals are indicated with vertical lines. **(C)** Brain *Pitpna* gene expression from *vibrator* mutant alleles was increased by E610G alleles. Measurements were made from animals in (A) not used in (B). Relative quantities compared to three reference genes (*Gapdh*, *Sdha*, and *Ppia*) are plotted. Individual points represent the average of three replicate measurements per biological sample.

The hypothesis that the E610G polymorphism is sufficient for the original modifier phenotype makes three explicit predictions: that the edited allele should suppress *vibrator* phenotypes relative to the background *Nxf1* ^*B6*^ (and silent, pseudo-edited) allele, that it should act semi-dominantly (and therefore be measurable in heterozygotes), and that it should be indistinguishable from the congenic *Nxf1* ^*CAST*^ allele in magnitude of effect. We examined 68 sequential *vibrator* (*Pitpn* ^*vb/vb*^) mutant and 47 control G2 animals in backcrosses of our edited alleles to the B6–*Pitpna* ^*vb*/+^, *Nxf1* ^*B6/CAST*^ stock (Fig. 5A). The E610G edited allele, but not the pseudo-edited control allele, altered the tremor phenotype of *vibrator* mutants–scored by observers blind to genotype–relative to the *Nxf1* ^*B6*^ allele (S1_Video and S2_Video). This held for heterozygotes in trans to the *Nxf1* ^*B6*^ allele (p = 7.9x10^-6^, Wilcoxon Rank Sum Test) and in trans to the *Nxf1* ^*CAST*^ allele (p = 2.0x10^-4^). The E610G edited allele was indistinguishable from the congenic *Nxf1* ^*CAST*^ allele in each context (Fig. 5A).

In a subset of animals followed for survival analysis, the edited allele prolonged the viability of *vibrator* mutants to the same degree as the original congenic allele, while the pseudo-edited allele did not (Fig. 5B). A Cox proportional hazards model showed significantly better survival with the edited allele (p = 0.0013) but not the pseudo-edited allele (p = 0.27) compared with the parental *Nxf1* ^*B6*^ allele when heterozygous with respect to *Nxf1* ^*B6*^. Similarly, among animals heterozygous to the congenic *Nxf1* ^*CAST*^ allele the observed deaths all occurred in unedited animals. Survival in unedited animals was different from non-mutant control and *Nxf1* ^*CAST*^ homozygotes (p = 0.046) under the proportional hazards model, while edited animals were not (p>0.99).

Most of the backcross animals were processed for gene expression analysis. The edited allele increased the expression of correctly spliced *Pitpna* mRNA from *vibrator* mutant alleles, again comparable to the congenic allele, as measured by qRT-PCR from brains of the same animals (Fig. 5C). Non-parametric tests supported significantly increased expression in each of the four predicted pair-wise comparisons and in each combination the edited allele was indistinguishable from the congenic allele. These data together show that the single amino acid substitution E610G is sufficient to account quantitatively for the previously observed *Modifier of-vibrator* (*Mvb1*) activities of the *Nxf1* ^*CAST*^ allele in vivo.

### *Nxf1* E610G edited allele is a general suppressor of IAP insertions

**Fig 6:**
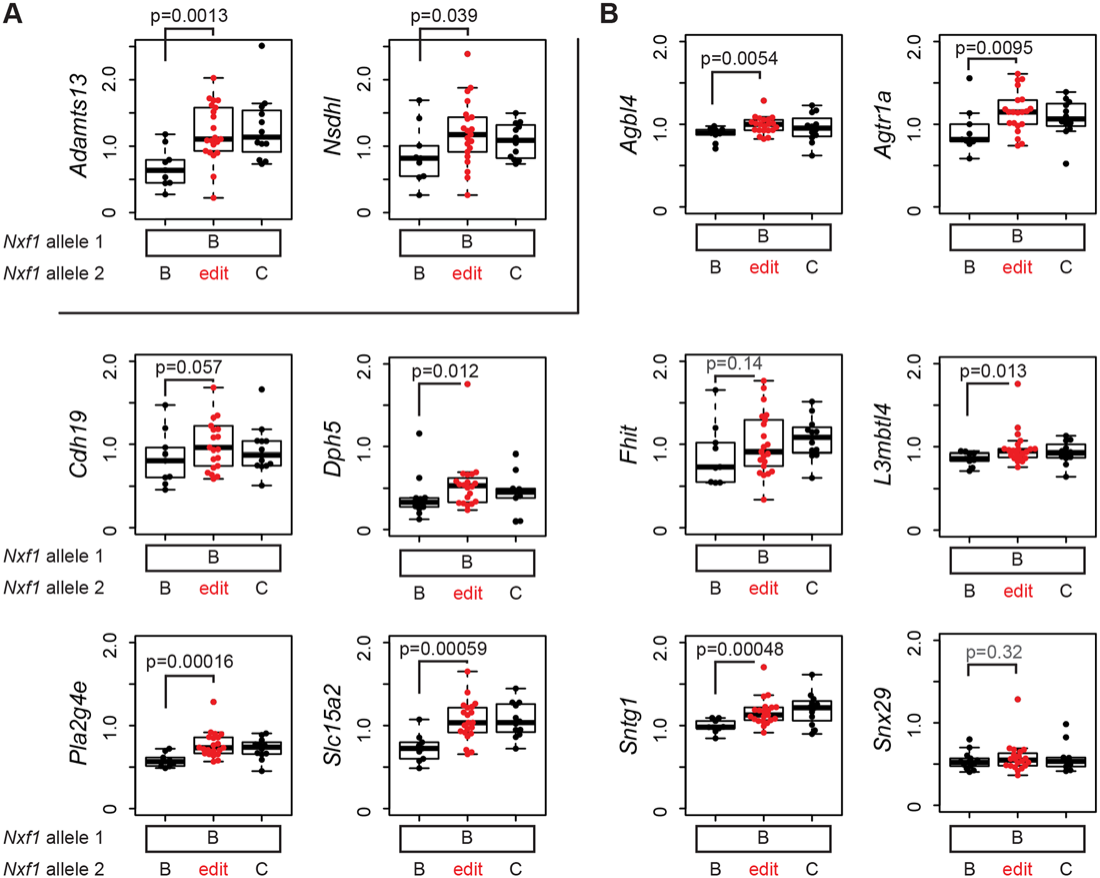
E610G replicates the modifier effect on IAP insertions in the B6 genome. **(A)** Relative expression levels of *Adamts13* and *Nsdhl* RNAs with the IAP-containing intron correctly spliced were measured by qRT-PCR from livers of animals heterozygous for the indicated *Nxf1* congenic (B, *Nxf1* ^*B6*^; C, *Nxf1* ^*CAST*^) or E610G edited allele. Expression measures are relative to five reference genes (*Desmin*, *Gapdh*, *Sdha*, *Ppia*, *Pitpna*) and divided by average relative value. Individual points represent the average of three replicate measurements per biological sample. **(B)** Similar measurements from brain cDNA of the same animals for the indicated genes. Measures are relative to four reference genes (*Gapdh*, *Sdha*, *Ppia*, *Pitpna*). P values are shown for a one-tailed Wilcoxon-Mann-Whitney test for the predicted increase in E610G edited animals relative to B6 homozygotes.

The discovery of B6-endogenous targets of the modifier activity in our original congenic stock (Figs. 1 and 2) predicts that if E610G amino acid substitution allele explains the modifier activity, it should act on expression level of those genes as well. We tested this prediction with 12 qRT-PCR assays on F1 samples that were heterozygous for the edited allele, but without potentially confounding *Pitpn* ^*vb*^ mutations (Fig. 6). Both *Adamts13* and *Nsdhl* showed suppression by the edited allele equivalent to the congenic allele in liver, where both are well-expressed (Fig. 6A). In brain cDNA, six of seven genes that were suppressed by the congenic stock (*Agbl4*, *Agtr1a*, *Cdh19*, *L3mbtl4*, *Pla2g4e*, and *Slc15a2*) showed effects of the heterozygous edited allele, while the seventh (*Fhit*) did not approach independent significance in the heterozygote samples (Fig. 6B). In addition, two of three genes for which the congenic experiments showed a trend but did not provide significant support (*Sntg1* and *Dph5*) also showed significant evidence of suppression by the edited allele (Fig. 6B). Each event survived correction for false discovery rate below or near q = 0.05 (S1 Table). The tested sites, including elements closely related to the non-suppressed insertion in *Atrn* ^*mgL*^, other full-length elements, IΔ1 elements, and other deletion classes, were significantly increased in a semi-dominant manner by the edited allele. Alternative processing events on chromosome 19 that differed between congenic stocks (Fig. 3C,D) were not significantly different between edited and unedited alleles (S4 Fig), confirming that they represented strain variations in each gene rather than effects of the modifier locus. Together, these measurements confirm that the E610G variant is able to suppress IAP-related gene expression changes at the wide array of sites detected for the congenic allele by our genome-wide screen and explains the modifier effect of previous congenic and transgenic strains [19, 20].

### Most of the IAP sequence is not required for mutation or suppression by *Nxf1*

We next asked whether identifiable sequence features correlated with expression level in inserted strains or response to *Nxf1* genotype. The ratio of expression in uninserted vs. inserted strains (U/I ratio) was nominally correlated with IAP length (Spearmen’s ρ = 0.32, p = 0.05) primarily due to lack of effect among LTR-only events and extreme values for *Adamts13* and *Zfp69* (S5 Fig). Intron size, flanking exon size, distance between the element and each exon, and annotation features of the exons for potential alternative events had no significant correlation.

The expression ratio between *Nxf1* ^*CAST*^ vs. *Nxf1* ^*B6*^ alleles (C/B ratio) was positively correlated with U/I ratio (Spearman’s ρ = 0.44, p = 0.0058). Intron length, distance from 5’ exon to the IAP, IAP length, length of each flanking exon, and whether 5’ or 3’ splice site showed evidence for alternative usage in UCSC annotations showed no significant correlation with the expression ratio between *Nxf1* alleles (S6 Fig). However, distance between IAP and 3’ exon approached a nominal significance threshold (ρ = -0.30, p = 0.051) before correction for multiple tests.

A simple alignment among elements that had shown both reduced expression in inserted strains and suppression by either *Nxf1* ^*CAST*^ or E610G alleles showed that IAP protein coding sequences were not required for either effect, leaving only LTRs, a small sequence 5’ to gag, ~60 bp in four small fragments within *pol*, and sequences 3’ to pol that include both RNA transport element (RTE) and the polypurine tract (ppt). For classification of elements as suppressed, we used Fisher’s combined probability test as a meta-analysis across the congenic homozygous and genome-edited heterozygous samples (S1 Table). We further characterized each element for presence and sequence divergence in eight segments: LTR, segment 2 (between LTR and *gag*), *gag* CDS, *prt* CDS, *pol* CDS, segment 6 (after pol), RTE, and polypurine tract. Phylogenetic trees for either full length or discreet segments of the IAP element did not correlate with either differences between inserted and uninserted strains or effect of *Nxf1* genotype (S7 Fig). Although suppressed insertions appear somewhat clustered with respect to RTE, identical RTE sequences were associated with divergent effects on host gene RNA (S7 Fig, panel J). Predicted RNA folding also did not show any strict requirement for suppression or lack of suppression by *Nxf1* alleles (S8 Fig). These data together show that most of the IAP consensus sequence is not required and suggest that a minimum element for both mutation and *Nxf1*-mediated suppression might require less than 2 kb (837 bp 5’ in *Cdh19* and 1108 bp 3’ in *Sntg1*), but that efficiency might be influenced by other factors–possibly including elements size, distance to the 3’ exon, and factors not readily predicted from sequence alone.

## Discussion

**Fig 7:**
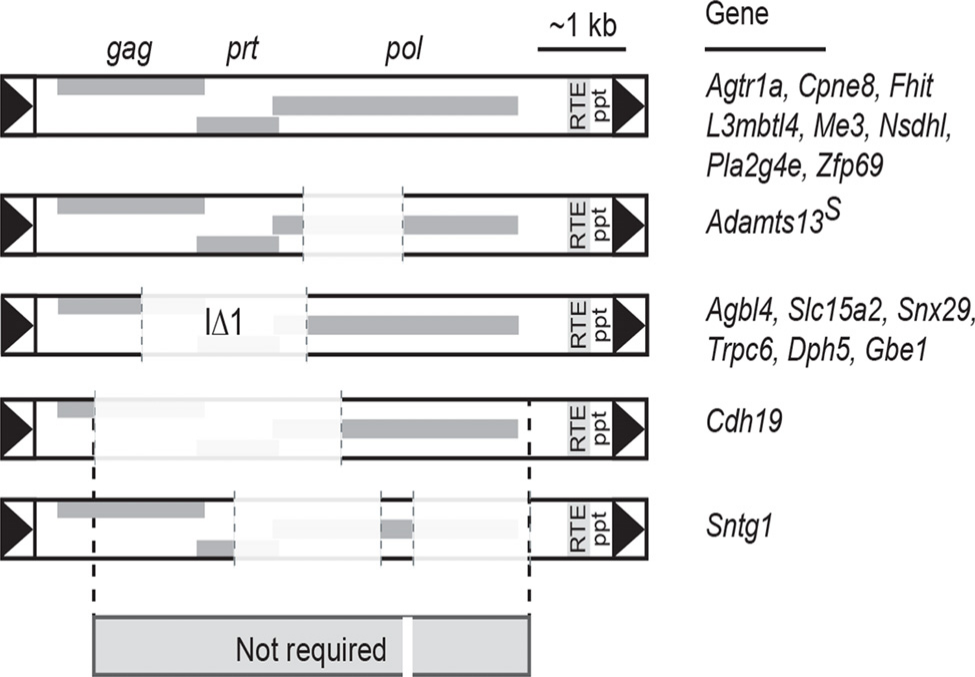
Shared sequence features of IAPs suppressed by *Nxf1* alleles. Alignment of IAPs whose effects on host gene expression were modified by either congenic or genome edited *Nxf1* alleles suggests that most of the IAP sequence is not required for either mutagenic potential or suppression by *Nxf1*. Shared sequence includes just 837 bp of LTR through 5’ end of the *gag* gene at *Cdh19*, ~60 bp in four fragments within *pol*, and 1108 bp including the RNA transport element (RTE), polypurine tract (ppt) and 3’ LTR.

As far as we are aware, *Nxf1* remains the most highly-connected node for genetic interactions among modifier genes that act on either natural or chemically-induced mutations in mice [19, 42]. The current results add three times as many allele-specific genetic interactions for *Mvb1* alleles of *Nxf1* (CAST and E610G) as had previously been reported. We previously showed that the most common *Mus musculus castaneus* allele of *Nxf1*, containing many polymorphic sites, suppressed the classical *vibrator* mutation [20] and that a congenic stock including this allele suppressed several other IΔ1 IAP insertional mutations [19]. Our results extend the diversity of IAP insertions susceptible to *Nxf1* substantially beyond the IΔ1 subfamily (Fig. 2) and provide further evidence against activity on alternative processing at non-IAP introns (Fig. 3). Alternative events suppressed by *Nxf1* alleles include known bleeding exons with alternative termination, such as *Slc15a2* [43] and *Trpc6* [30], and IAP-dependent alternative splicing, such as *Adamts13* [27, 44] and *Cpne8*, as well as introns for which public transcriptome data do not indicate a predominant alternative variant. Comparison of IAP elements whose effects can be suppressed by *Nxf1* alleles further suggests minimum sequence requirements for both mutation and suppression lie in <1 kb in the 5’ end and ~1 kb in the 3’ end of the IAP (Fig. 7), although construction and experimental measurements of a minimal element will be required to test this idea.

In contrast to most de novo mutations caused by IAP insertions, the magnitude of gene expression changes is relatively modest for most of the B6-endogenous IAP sites we tested (Fig. 2). Indeed, recent evaluation of mouse transposable elements concluded that insertion events that strongly affected gene expression were rapidly purged from populations, hence remaining elements tend toward more modest effect sizes [45]. *Adamts13* is a notable exception, with ~1000-fold change in completion of the transcript across the inserted intron (Fig. 1). However, as the truncated Adamts13 encoded by the major B6 transcript retains substantial function [44, 46] and the absolute level of full-length transcript recovered by suppression is modest, little physiological consequence is expected from this increase in full-length Adamts13 protein above the level of function provided by the abundant truncated form. Taken all together the data suggest that using IAP insertional mutations in combination with *Nxf1* suppressing alleles should allow relatively clean titrations of intermediate gene doses. This may have application in modeling genetic disorders (and therapeutics) in mice.

Our results using genome-edited mice provide molecular precision to the definition of the modifier allele. Although several polymorphisms distinguish *Nxf1* ^*B6*^ and *Nxf1* ^*CAST*^ alleles in both transgenic [20] and congenic [19] experiments, the E610G single amino acid substitution (Fig. 4) was sufficient for the full range of genetic modifier activity previously ascribed to the congenic locus and published 16 kb transgene. The point mutation modified all of the principle features of the *vibrator* mutation on which the modifier activity was first described [47] with the characteristic semi-dominant effect (Fig. 5). The ability to assay the edited allele as a heterozygote with respect to congenic *Nxf1* ^*B6*^ and *Nxf1* ^*CAST*^ alleles and the specificity of multiple phenotypes assayed (Figs. 5, 6) also provides an additional safeguard against potential off-target modifications in the editing process.

Our results demonstrate that a single amino acid substitution in the C-terminal, UBA-like domain of Nxf1 simultaneously enhances expression of full-length mRNA at more than a dozen loci in the mouse reference genome at the level of pre-mRNA processing. This illustrates a potential post-transcriptional mechanism for evolution of gene regulatory networks selected from events generated by molecular parasites [3] and provides a post-transcriptional tool for modulating expression level of any mouse gene into which an IAP element can be introduced.

## Methods

### Ethics statement

All animal procedures were approved by the University of California, San Diego Institutional Animal Care and Use Committee (IACUC). Tissues were obtained after terminal anesthesia with tribromoethanol (avertin)

### Mice

Congenic C57BL/6J (B6)–*Nxf1* ^*CAST*^ mice were developed and maintained in our laboratory by serial backcross and genotyped by PCR as previously described [19]. Mice in this report were at backcross generation N30 or later. A/J, BALB/cJ and 129S1/SvImJ were purchased from The Jackson Laboratory and bred locally in the same vivarium room with our congenic stocks. *Adamts13* genotypes were determined by PCR using a three primer combination: ACCTCTCAAGTGTTTGGGATGCTA, TCAGCGCCATCTTGTGACGGCGAA, and TGCCAGATGGCCATGATTAACTCT [27]. Tissues were from paired F2 animals from a B6 x BALB/c intercross previously described [19] (Fig. 1C) or B6 congenic or co-isogenic stocks (all others). Five male and five female pairs were used for measurements from congenic animals. No sex-specific differences were noted. Genome-edited mice were produced in the Moores UCSD Cancer Center Transgenic Mouse Shared Resource by co-injection of mouse C57BL/6 one-cell embryos with sgRNA, Cas9 mRNA, and unpurified oligonucleotide as a homology-dependent repair donor essentially as described [40]. Oligonucleotide sequences for sgRNA, Cas9 amplification, and homology dependent repair are given in S2 Table. Measurements from genome edited mice used unpaired samples collected as a cohort and comprising ~equal numbers of each sex.

### Identification of B6 alleles with IAP element insertions

Two independent approaches identified intronic IAP elements. IAP annotations from the Repbase Update definitions were mined from the RepeatMasker track data table in the UCSC Genome Browser (S1 Fig). A custom table was also developed by iterative BLAT searches with a panel of short (35 bp) sequences conserved in subfamilies of IAP elements for which we had previously observed *Nxf1*-related suppression (S3 Table). Both sets were filtered by strand orientation in Galaxy [48] and compared to UCSC intron or RefSeq gene boundary annotations. After manual curation to remove likely annotation errors, the oligonucleotide-derived set identified 70 and the Repbase Update annotations identified a superset of 85 IAP-family elements within well-defined introns or 3’ ends that were premature relative to human or rat orthologs. Alignments of IAP sequences were performed in MUSCLE (http://www.ebi.ac.uk/Tools/msa/muscle/) using default parameters.

### Quantitative RT-PCR assays

PCR assays for transcripts that are correctly spliced around the IAP insertion and for reference genes were designed relative to exon junctions using Primer3 [49]. Assays were selected for use if they produced a single product by melt profile, single band by gel imaging, and low coefficient of variation among replicate samples. Specific primers used are provided in S4 Table. RNA was prepared from tissues homogenized in Trizol, converted to cDNA by reverse transcription, and assayed by quantitative PCR using SYBR green fluorescence in a Bio-Rad CFX instrument as previously described [19]. Normalization was performed by geometric averaging among the indicated reference genes, as described [50], and implemented in software supplied by the manufacturer. Four reference gene assays, *Gapdh*, *Ppia*, *Sdha*, and *Pitpna* were selected based on high cycle efficiencies, accurate dose titration, and low quantitative variation across tested conditions (only the first three were used for comparisons including *vibrator* samples, which express ~18% normal level of *Pitpna*).

### Gel-based assays

Relative quantification of alternative splicing products was carried out by gel electrophoresis of products from non-saturating 3-primer competitive PCR amplifications based on pilot experiments for each assay. Samples for comparison were amplified side-by-side and resolved on the same gel. Relative fluorescence intensities of resolved bands were quantified from non-saturated. tiff image files using ImageJ software.

### Splicing-sensitive array profiling

Affymetrix MJAY microarrays (GEO platform GPL13185) were hybridized and processed as described [35, 39], for brain RNA from 3 pairs of littermates (two female pairs, one male pair) with opposite *Nxf1* genotypes. The separation score was defined as the log_2_ (ratio of alternative events in *Nxf1* ^*CAST*^ samples / ratio of alternative events in *Nxf1* ^*B6*^ samples) [36]. Events with a separation score >0.5 (~1.4-fold change) and false discovery rate q<0.05 were deemed significant.

### Statistics

Statistical tests were conducted in R. Non-parametric rank tests were performed in package exactRankTests or coin; both packages gave identical results for repeated analyses. Non-parametric correlation tests with ties were performed in the package Hmisc. Correlation analyses and standard hypothesis tests were conducted in R 2.8.1 or later running on Mac OS 10.5.8 or later.

## Supporting Information

S1 Table False discovery rates and meta-analyses. Non-parametric p-values for the effect of *Nxf1* alleles were corrected for false discovery rate (FDR) using the Benjamini-Hochberg procedure within each IAP class and experimental group (congenic stocks or genome edited allele). For sites measured in both experiments meta-analysis was performed using Fisher’s combined probability test. All tests were conducted in R.
(XLSX)

S2 Table Oligonucleotides used for *Nxf1* E610G genome editing and validation. Sequences are presented for production of single guide RNAs and synthetic Cas9 mRNA, for single-stranded donor templates for homology-dependent repair, and for sequencing target and predicted off-target cleavage sites for the guide RNA used in genome editing.
(XLSX)

S3 Table IAP seed sequences. Sequences used in genome searches for IAP sequences in introns.
(XLSX)

S4 Table PCR primers used to assay RNA abundance or isoform variation. Primer sequences are given, by class of assay, for dye intercalation quantitative PCR (Fig. 2B and Fig. 6), cassette exon inclusion and alternative splice site usage (Fig. 3A), and alternative events predicted by microarray data (Fig. 3C).
(XLSX)

S1 Fig Galaxy workflow to identify intronic IAPs. Mouse reference assembly mm9 was accessed on 1/15/12 and analyzed in Galaxy using the workflow as illustrated. Numbers in parentheses indicate the number of objects contained in each product after each operation. Operations to identify strand specificity are color coded.
(TIF)

S2 Fig Assay development and quality control workflow. The number of successes and attempts in each step are indicated. PCR primers were selected in Primer3 web interface (http://bioinfo.ut.ee/primer3-0.4.0/primer3/input.htm). Both melt profile after real-time PCR and gel electrophoresis were used to determine that each PCR produced a single significant product. Coefficient of variation after normalization to reference genes was used to eliminate assays with limited power to detect differences by genotype. Gene assays whose measurements differed significantly by *Nxf1* genotype in the congenic strains are shown in red. Inequality symbols in the lower boxes indicate groups with greater or lesser levels of correctly processed RNA based on qRT-PCR assays described in Fig. 2 and accompanying text.
(TIF)

S3 Fig *Nxf1* alleles do not affect alternative splicing at selected non-IAP splice choices. **(A)** Gel assays for well-characterized alternative splicing of cassette exons, including several encoding Nxf1-interacting factors, show no difference in general or tissue-specific inclusion rates between *Nxf1* ^*B6*^ (B) and *Nxf1* ^*CAST*^ (C) alleles in congenic mice. Splice choice is diagrammed, with PCR product sizes indicated. Bar graphs show proportion of fluorescence intensity in each band among 13–20 independent sample pairs tested for each gene. **(B)** Single examples for a retained intron (*Ank1*), alternative splice acceptor (*Nup214*) and alternative splice donor (*Nup98*) show no difference between *Nxf1* alleles.
(TIF)

S4 Fig Alternative splicing in the congenic interval is not explained by E610G. The alternative events in the congenic interval on chromosome 19 that were found by the arrays and confirmed in gel assays were re-assayed in co-isogenic E610G genome-edited animals and controls, using the samples from Fig. 6 and the assays from Fig. 3. *Trpc6* gel assay, included as a positive control, showed suppression of the IAP-dependent splice form. None of the four chromosome 19 alternative events that were significant in both array (Fig. 3B) and gel (Fig. 3C) assays showed significant differences between edited and unedited heterozygotes with respect to *Nxf1* ^*B6*^. Indicated p-vlaues are for the one-tailed Wilcoxon rank sum test for the direction of difference seen in Fig. 3C, with the exception of *Abca5*, included as a negative control, which shows the value for the two-tailed test, as it had no predicted difference. Inclusion of heterozygous congenic samples confirms the effect, detection sensitivity and allele-independents effects predicted from Fig. 3C for *Ganab* (p = 0.00015) and *Tmem138* (p = 0.0036), but not for *Fkbp2* (p = 0.28) or *Stx3* (p = 0.40), albeit comparing new edited with archived congenic samples.
(TIF)

S5 Fig Lack of strong correlations between genomic context and IAP-associated expression changes. Plots show relationships between indicated attributes of the studied introns (x-axes) and expression ratio between uninserted strain and inserted strains with respect to the IAP (U/I ratio). U/I ratio is expressed as log10 to permit inclusion of exceptionally strong effects at *Adamts13* and *Zfp69*. Dashed line at zero indicates elements with no measureable effect. Spearman correlation coefficients (ρ) and associated p-values are shown. For categorical annotations of alternatively spliced (alt), alternative 3’ end formation (altEnd), constitutive (con), and 3’ terminal (end) exons, p-values are derived from the Kruskal-Wallis test. The magnitude of U/I ratio is moderately well correlated with its p-value, as expected for events near the threshold for discrimination and in the presence of other potential modifying effects across strains.
(TIF)

S6 Fig Lack of strong correlations between genomic features and *Nxf1*-dependent suppression. Plots show relationships between indicated attributes of the studied introns (x-axes) and expression ratio between *Nxf1* ^*CAST*^ and *Nxf1* ^*B6*^ alleles (C/B ratio). For categorical annotations of alternatively spliced (alt), alternative 3’ end formation (altEnd), constitutive (con), and 3’ terminal (end) exons, p-values are derived from the Kruskal-Wallis test. Nominal evidence for correlation to distance from 3’ exon does not survive correction for multiple tests, but may merit further study. As expected, C/B ratio is correlated with U/I ratio, as larger mutational effects should provide a larger phenotypic space for suppression.
(TIF)

S7 Fig Phylogenetic relationships among IAP elements. Neighbor-joining trees were generated from alignments in MUSCLE (http://www.ebi.ac.uk/Tools/msa/muscle/) for the indicated IAP groups or sequence components. Elements are colored as in Fig. 2, red for those with strong evidence for *Nxf1*-mediated suppression, grey for those with no evidence of mutagenic effect across strains. Nucleotide length of the total alignment, as well as lengths of the median, shortest and longest input sequences are indicated for each class or segment of IAP sequence. (**A**) Full alignment of IAP sequences >7kb (“full length”). (**B**) Full alignment of elements containing the IΔ1 deletion. Alignments of specific sequence components are (**C**) LTRs, (**D**) interval between LTR and *gag*, (E) *gag*, (**F**) *prt*, (**G**) *prt* to *pol*, (**H**) *pol*, (**I**) *pol* to the RTE, (**J**) RTE, (**K**) ppt.
(PDF)

S8 Fig RTE secondary structure does not determine *Nxf1*-dependent suppression. Secondary structures of RTE RNAs were predicted using the mfold web server version 3.4 (http://mfold.rna.albany.edu/?q=mfold/download-mfold) under default parameters. **(A)** RTE phylogeny, as in S7 Fig, panel J. **(B)** Optimal (left column) and next best (right column) predicted secondary structures of RTE RNA sequence for each unique phylogeny group, based on calculated lowest free energy, are depicted. **(C)** Free energy of optimal structures, and **(D)** difference in free energy between optimal and next best secondary structures are listed. In addition to identical RTE sequences with divergent outcomes, RTE structures from adjacent positions in the tree also showed dissimilar effects (e.g., *hes*, *mgL*, *Nudcd3*, and *Nsdhl*).
(TIF)

S1 Video *Vibrator* mutant with silent edit only. Moderate tremor of this *Pitpna* ^*vb/vb*^ *Nxf1* ^*pseudo/CAST*^ animal is characteristic of *Pitpna* ^*vb/vb*^ in the presence of heterozygous *Nxf1* ^*B6/CAST*^, consistent with no effect of the “pseudo-edited” allele that altered only the silent site within the sgRNA target sequence.
(MP4)

S2 Video *Vibrator* mutant with E610G edited allele. Mild tremor seen in this *Pitpna* ^*vb/vb*^ *Nxf1* ^*E610G/CAST*^ animal is characteristic of of *Pitpna* ^*vb/vb*^ in the presence of homozygous *Nxf1* ^*CAST/CAST*^, consistent with quantitative effect of the E610G edited allele reproducing the original *Modifier-of-vibrator* effect.
(MP4)

## References

[1] Cowley M, Oakey RJ. Transposable elements re-wire and fine-tune the transcriptome. PLoS genetics. 2013;9(1):e1003234 Epub 2013/01/30. 10.1371/journal.pgen.1003234 23358118 PMC3554611

[2] Gifford WD, Pfaff SL, Macfarlan TS. Transposable elements as genetic regulatory substrates in early development. Trends Cell Biol. 2013;23(5):218–26. Epub 2013/02/16. 10.1016/j.tcb.2013.01.001 23411159 PMC4034679

[3] Peter IS, Davidson EH. Evolution of gene regulatory networks controlling body plan development. Cell. 2011;144(6):970–85. Epub 2011/03/19. 10.1016/j.cell.2011.02.017 21414487 PMC3076009

[4] Waterston RH, Lindblad-Toh K, Birney E, Rogers J, Abril JF, Agarwal P, et al Initial sequencing and comparative analysis of the mouse genome. Nature. 2002;420(6915):520–62. 12466850 10.1038/nature01262

[5] Hamilton BA, Frankel WN. Of mice and genome sequence. Cell. 2001;107(1):13–6. 11595181 10.1016/s0092-8674(01)00514-1

[6] Maksakova IA, Romanish MT, Gagnier L, Dunn CA, van de Lagemaat LN, Mager DL. Retroviral elements and their hosts: insertional mutagenesis in the mouse germ line. PLoS genetics. 2006;2(1):e2 16440055 10.1371/journal.pgen.0020002 PMC1331978

[7] Vasicek TJ, Zeng L, Guan XJ, Zhang T, Costantini F, Tilghman SM. Two dominant mutations in the mouse fused gene are the result of transposon insertions. Genetics. 1997;147(2):777–86. 9335612 10.1093/genetics/147.2.777 PMC1208197

[8] Duhl DM, Vrieling H, Miller KA, Wolff GL, Barsh GS. Neomorphic agouti mutations in obese yellow mice. Nature Genet. 1994;8(1):59–65. 7987393 10.1038/ng0994-59

[9] Lugani F, Arora R, Papeta N, Patel A, Zheng Z, Sterken R, et al A retrotransposon insertion in the 5' regulatory domain of Ptf1a results in ectopic gene expression and multiple congenital defects in Danforth's short tail mouse. PLoS genetics. 2013;9(2):e1003206 Epub 2013/02/26. 10.1371/journal.pgen.1003206 23437001 PMC3578747

[10] Semba K, Araki K, Matsumoto K, Suda H, Ando T, Sei A, et al Ectopic expression of Ptf1a induces spinal defects, urogenital defects, and anorectal malformations in Danforth's short tail mice. PLoS genetics. 2013;9(2):e1003204 Epub 2013/02/26. 10.1371/journal.pgen.1003204 23436999 PMC3578775

[11] Vlangos CN, Siuniak AN, Robinson D, Chinnaiyan AM, Lyons RH Jr., Cavalcoli JD, et al Next-generation sequencing identifies the Danforth's short tail mouse mutation as a retrotransposon insertion affecting Ptf1a expression. PLoS genetics. 2013;9(2):e1003205 Epub 2013/02/26. 10.1371/journal.pgen.1003205 23437000 PMC3578742

[12] Bentley DL. Coupling mRNA processing with transcription in time and space. Nat Rev Genet. 2014;15(3):163–75. Epub 2014/02/12. 10.1038/nrg3662 24514444 PMC4304646

[13] Auboeuf D, Honig A, Berget SM, O'Malley BW. Coordinate regulation of transcription and splicing by steroid receptor coregulators. Science. 2002;298(5592):416–9. 12376702 10.1126/science.1073734

[14] Cramer P, Pesce CG, Baralle FE, Kornblihtt AR. Functional association between promoter structure and transcript alternative splicing. Proc Natl Acad Sci U S A. 1997;94(21):11456–60. Epub 1997/10/23. 9326631 10.1073/pnas.94.21.11456 PMC23504

[15] Kornblihtt AR. Promoter usage and alternative splicing. Curr Opin Cell Biol. 2005;17(3):262–8. 15901495 10.1016/j.ceb.2005.04.014

[16] de la Mata M, Kornblihtt AR. RNA polymerase II C-terminal domain mediates regulation of alternative splicing by SRp20. Nature structural & molecular biology. 2006;13(11):973–80. 10.1038/nsmb1155 17028590

[17] Kornblihtt AR. Chromatin, transcript elongation and alternative splicing. Nature structural & molecular biology. 2006;13(1):5–7. 10.1038/nsmb0106-5 16395314

[18] Vargas DY, Shah K, Batish M, Levandoski M, Sinha S, Marras SA, et al Single-molecule imaging of transcriptionally coupled and uncoupled splicing. Cell. 2011;147(5):1054–65. Epub 2011/11/29. 10.1016/j.cell.2011.10.024 22118462 PMC3245879

[19] Concepcion D, Flores-Garcia L, Hamilton BA. Multipotent genetic suppression of retrotransposon-induced mutations by Nxf1 through fine-tuning of alternative splicing. PLoS genetics. 2009;5(5):e1000484 10.1371/journal.pgen.1000484 19436707 PMC2674570

[20] Floyd JA, Gold DA, Concepcion D, Poon TH, Wang X, Keithley E, et al A natural allele of Nxf1 suppresses retrovirus insertional mutations. Nature Genetics. 2003;35:221–8. 14517553 10.1038/ng1247 PMC2756099

[21] Kuff EL, Lueders KK. The intracisternal A-particle gene family: structure and functional aspects. Advances in cancer research. 1988;51:183–276. 3146900 10.1016/s0065-230x(08)60223-7

[22] Fujikawa K, Suzuki H, McMullen B, Chung D. Purification of human von Willebrand factor-cleaving protease and its identification as a new member of the metalloproteinase family. Blood. 2001;98(6):1662–6. 11535495 10.1182/blood.v98.6.1662

[23] Gerritsen HE, Robles R, Lammle B, Furlan M. Partial amino acid sequence of purified von Willebrand factor-cleaving protease. Blood. 2001;98(6):1654–61. 11535494 10.1182/blood.v98.6.1654

[24] Levy GG, Nichols WC, Lian EC, Foroud T, McClintick JN, McGee BM, et al Mutations in a member of the ADAMTS gene family cause thrombotic thrombocytopenic purpura. Nature. 2001;413(6855):488–94. 11586351 10.1038/35097008

[25] Nolasco LH, Turner NA, Bernardo A, Tao Z, Cleary TG, Dong JF, et al Hemolytic uremic syndrome-associated Shiga toxins promote endothelial-cell secretion and impair ADAMTS13 cleavage of unusually large von Willebrand factor multimers. Blood. 2005;106(13):4199–209. 16131569 10.1182/blood-2005-05-2111 PMC1895236

[26] Fujioka M, Hayakawa K, Mishima K, Kunizawa A, Irie K, Higuchi S, et al ADAMTS13 gene deletion aggravates ischemic brain damage: a possible neuroprotective role of ADAMTS13 by ameliorating postischemic hypoperfusion. Blood. 2010;115(8):1650–3. 10.1182/blood-2009-06-230110 19965676

[27] Banno F, Kaminaka K, Soejima K, Kokame K, Miyata T. Identification of strain-specific variants of mouse Adamts13 gene encoding von Willebrand factor-cleaving protease. J Biol Chem. 2004;279(29):30896–903. 15136581 10.1074/jbc.M314184200

[28] Uemura M, Tatsumi K, Matsumoto M, Fujimoto M, Matsuyama T, Ishikawa M, et al Localization of ADAMTS13 to the stellate cells of human liver. Blood. 2005;106(3):922–4. 15855280 10.1182/blood-2005-01-0152

[29] Zhou W, Inada M, Lee TP, Benten D, Lyubsky S, Bouhassira EE, et al ADAMTS13 is expressed in hepatic stellate cells. Laboratory investigation; a journal of technical methods and pathology. 2005;85(6):780–8. 15806136 10.1038/labinvest.3700275 PMC2573995

[30] Zhang Y, Romanish MT, Mager DL. Distributions of transposable elements reveal hazardous zones in mammalian introns. PLoS Comput Biol. 2011;7(5):e1002046 Epub 2011/05/17. 10.1371/journal.pcbi.1002046 21573203 PMC3088655

[31] Zhang Y, Maksakova IA, Gagnier L, van de Lagemaat LN, Mager DL. Genome-Wide Assessments Reveal Extremely High Levels of Polymorphism of Two Active Families of Mouse Endogenous Retroviral Elements. PLoS genetics. 2008;4(2):e1000007 10.1371/journal.pgen.1000007 18454193 PMC2265474

[32] Yalcin B, Wong K, Agam A, Goodson M, Keane TM, Gan X, et al Sequence-based characterization of structural variation in the mouse genome. Nature. 2011;477(7364):326–9. Epub 2011/09/17. 10.1038/nature10432 21921916 PMC3428933

[33] Keane TM, Goodstadt L, Danecek P, White MA, Wong K, Yalcin B, et al Mouse genomic variation and its effect on phenotypes and gene regulation. Nature. 2011;477(7364):289–94. Epub 2011/09/17. 10.1038/nature10413 21921910 PMC3276836

[34] Barash Y, Calarco JA, Gao W, Pan Q, Wang X, Shai O, et al Deciphering the splicing code. Nature. 2010;465(7294):53–9. 10.1038/nature09000 20445623

[35] Huelga SC, Vu AQ, Arnold JD, Liang TY, Liu PP, Yan BY, et al Integrative genome-wide analysis reveals cooperative regulation of alternative splicing by hnRNP proteins. Cell reports. 2012;1(2):167–78. Epub 2012/05/11. 10.1016/j.celrep.2012.02.001 22574288 PMC3345519

[36] Du H, Cline MS, Osborne RJ, Tuttle DL, Clark TA, Donohue JP, et al Aberrant alternative splicing and extracellular matrix gene expression in mouse models of myotonic dystrophy. Nature structural & molecular biology. 2010;17(2):187–93. Epub 2010/01/26. 10.1038/nsmb.1720 PMC2852634 20098426

[37] Gehman LT, Stoilov P, Maguire J, Damianov A, Lin CH, Shiue L, et al The splicing regulator Rbfox1 (A2BP1) controls neuronal excitation in the mammalian brain. Nat Genet. 2011;43(7):706–11. Epub 2011/05/31. 10.1038/ng.841 21623373 PMC3125461

[38] Lagier-Tourenne C, Polymenidou M, Hutt KR, Vu AQ, Baughn M, Huelga SC, et al Divergent roles of ALS-linked proteins FUS/TLS and TDP-43 intersect in processing long pre-mRNAs. Nature neuroscience. 2012;15(11):1488–97. Epub 2012/10/02. 10.1038/nn.3230 23023293 PMC3586380

[39] Polymenidou M, Lagier-Tourenne C, Hutt KR, Huelga SC, Moran J, Liang TY, et al Long pre-mRNA depletion and RNA missplicing contribute to neuronal vulnerability from loss of TDP-43. Nature neuroscience. 2011;14(4):459–68. Epub 2011/03/02. 10.1038/nn.2779 21358643 PMC3094729

[40] Wang H, Yang H, Shivalila CS, Dawlaty MM, Cheng AW, Zhang F, et al One-step generation of mice carrying mutations in multiple genes by CRISPR/Cas-mediated genome engineering. Cell. 2013;153(4):910–8. Epub 2013/05/07. 10.1016/j.cell.2013.04.025 23643243 PMC3969854

[41] Ran FA, Hsu PD, Wright J, Agarwala V, Scott DA, Zhang F. Genome engineering using the CRISPR-Cas9 system. Nature protocols. 2013;8(11):2281–308. Epub 2013/10/26. 10.1038/nprot.2013.143 24157548 PMC3969860

[42] Hamilton BA, Yu BD. Modifier genes and the plasticity of genetic networks in mice. PLoS genetics. 2012;8(4):e1002644 Epub 2012/04/19. 10.1371/journal.pgen.1002644 22511884 PMC3325199

[43] Li J, Akagi K, Hu Y, Trivett AL, Hlynialuk CJ, Swing DA, et al Mouse endogenous retroviruses can trigger premature transcriptional termination at a distance. Genome Res. 2012;22(5):870–84. Epub 2012/03/01. 10.1101/gr.130740.111 22367191 PMC3337433

[44] Zhou W, Bouhassira EE, Tsai HM. An IAP retrotransposon in the mouse ADAMTS13 gene creates ADAMTS13 variant proteins that are less effective in cleaving von Willebrand factor multimers. Blood. 2007;110(3):886–93. 17426255 10.1182/blood-2007-01-070953 PMC1924774

[45] Nellaker C, Keane TM, Yalcin B, Wong K, Agam A, Belgard TG, et al The genomic landscape shaped by selection on transposable elements across 18 mouse strains. Genome Biol. 2012;13(6):R45 Epub 2012/06/19. 10.1186/gb-2012-13-6-r45 22703977 PMC3446317

[46] Banno F, Chauhan AK, Kokame K, Yang J, Miyata S, Wagner DD, et al The distal carboxyl-terminal domains of ADAMTS13 are required for regulation of in vivo thrombus formation. Blood. 2009;113(21):5323–9. 10.1182/blood-2008-07-169359 19109562 PMC2686194

[47] Hamilton BA, Smith DJ, Mueller KL, Kerrebrock AW, Bronson RT, van Berkel V, et al The vibrator mutation causes neurodegeneration via reduced expression of PITP alpha: positional complementation cloning and extragenic suppression. Neuron. 1997;18(5):711–22. 9182797 10.1016/s0896-6273(00)80312-8

[48] Giardine B, Riemer C, Hardison RC, Burhans R, Elnitski L, Shah P, et al Galaxy: a platform for interactive large-scale genome analysis. Genome Res. 2005;15(10):1451–5. Epub 2005/09/20. 10.1101/gr.4086505 16169926 PMC1240089

[49] Rozen S, Skaletsky H. Primer3 on the WWW for general users and for biologist programmers. Methods in molecular biology (Clifton, NJ. 2000;132:365–86. 10547847 10.1385/1-59259-192-2:365

[50] Vandesompele J, De Preter K, Pattyn F, Poppe B, Van Roy N, De Paepe A, et al Accurate normalization of real-time quantitative RT-PCR data by geometric averaging of multiple internal control genes. Genome biology. 2002;3(7):RESEARCH0034 Epub 2002/08/20. 12184808 10.1186/gb-2002-3-7-research0034 PMC126239

