# Supplementary figures and images for: Nxf1 Natural Variant E610G Is a Semi-dominant Suppressor of IAP-Induced RNA Processing Defects
Source: PLoS Genet. 2015 Apr 2;11(4):e1005123. doi: 10.1371/journal.pgen.1005123 (PMC4383553; doi:10.1371/journal.pgen.1005123)

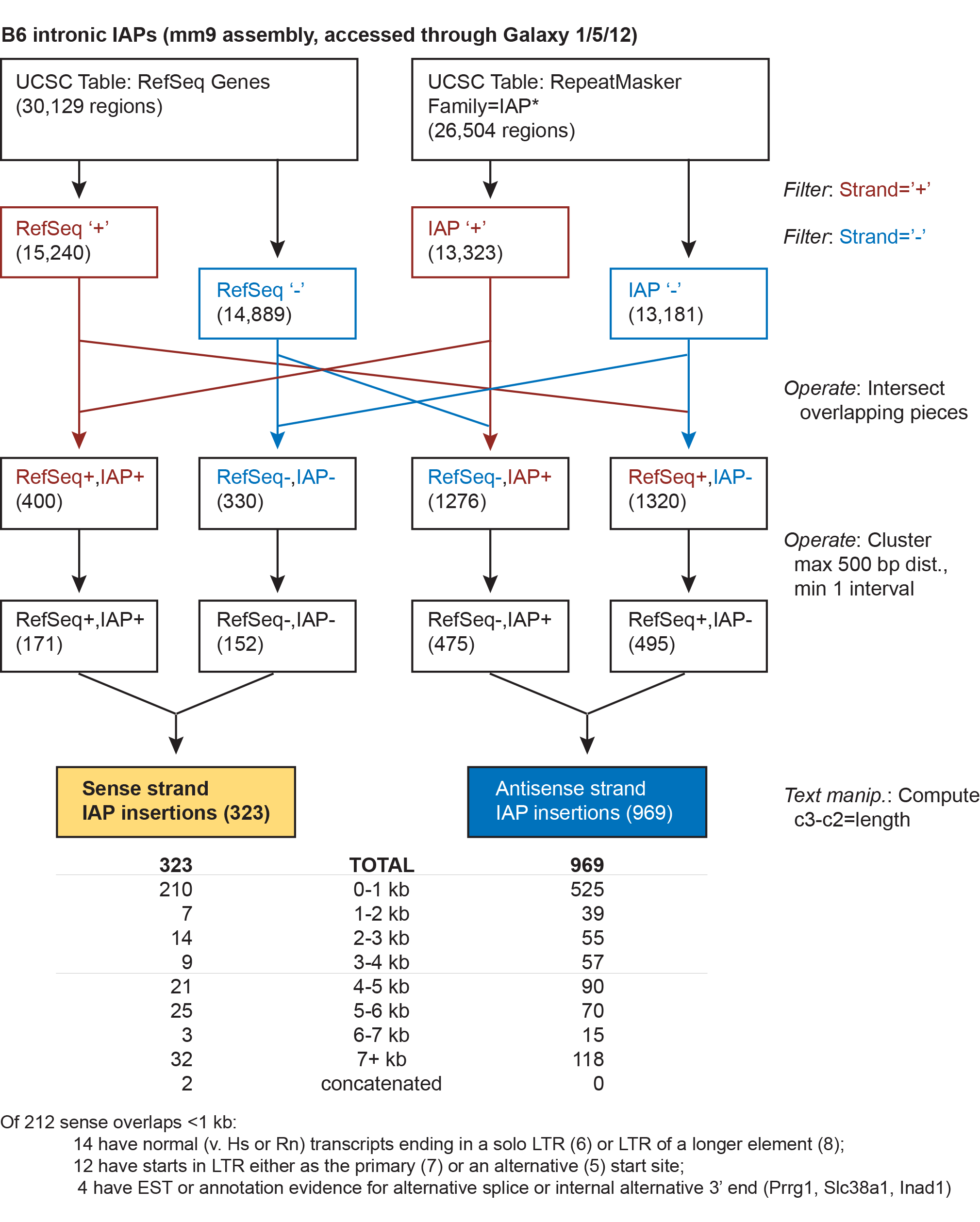

Supplement: S1 Fig — Mouse reference assembly mm9 was accessed on 1/15/12 and analyzed in Galaxy using the workflow as illustrated. Numbers in parentheses indicate the number of objects contained in each product after each operation. Operations to identify strand specificity are color coded. (TIF) [file pgen.1005123.s005.tif]

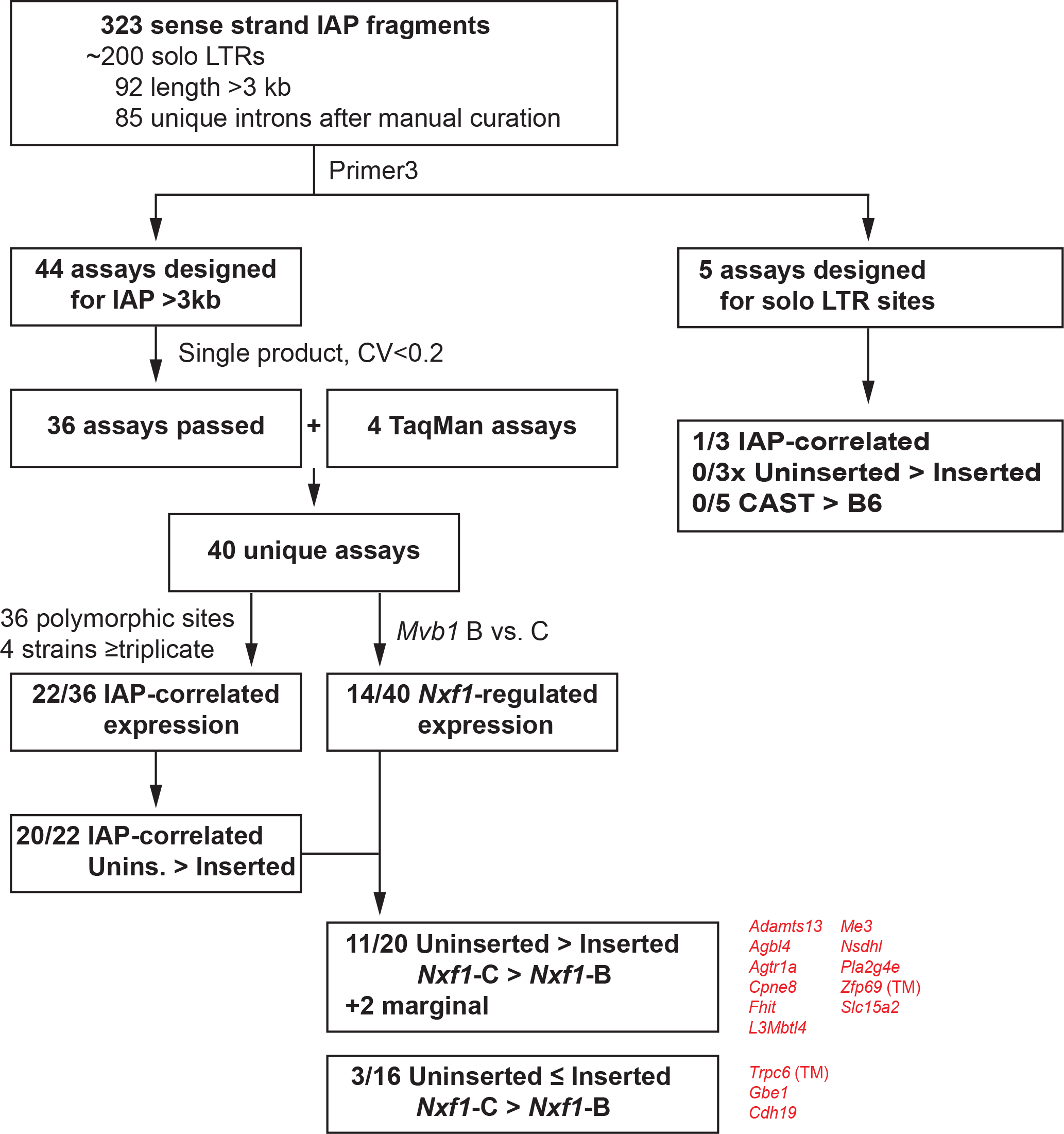

Supplement: S2 Fig — The number of successes and attempts in each step are indicated. PCR primers were selected in Primer3 web interface (http://bioinfo.ut.ee/primer3-0.4.0/primer3/input.htm). Both melt profile after real-time PCR and gel electrophoresis were used to determine that each PCR produced a single significant product. Coefficient of variation after normalization to reference genes was used to eliminate assays with limited power to detect differences by genotype. Gene assays whose measurements differed significantly by Nxf1 genotype in the congenic strains are shown in red. Inequality symbols in the lower boxes indicate groups with greater or lesser levels of correctly processed RNA based on qRT-PCR assays described in Fig. 2 and accompanying text. (TIF) [file pgen.1005123.s006.tif]

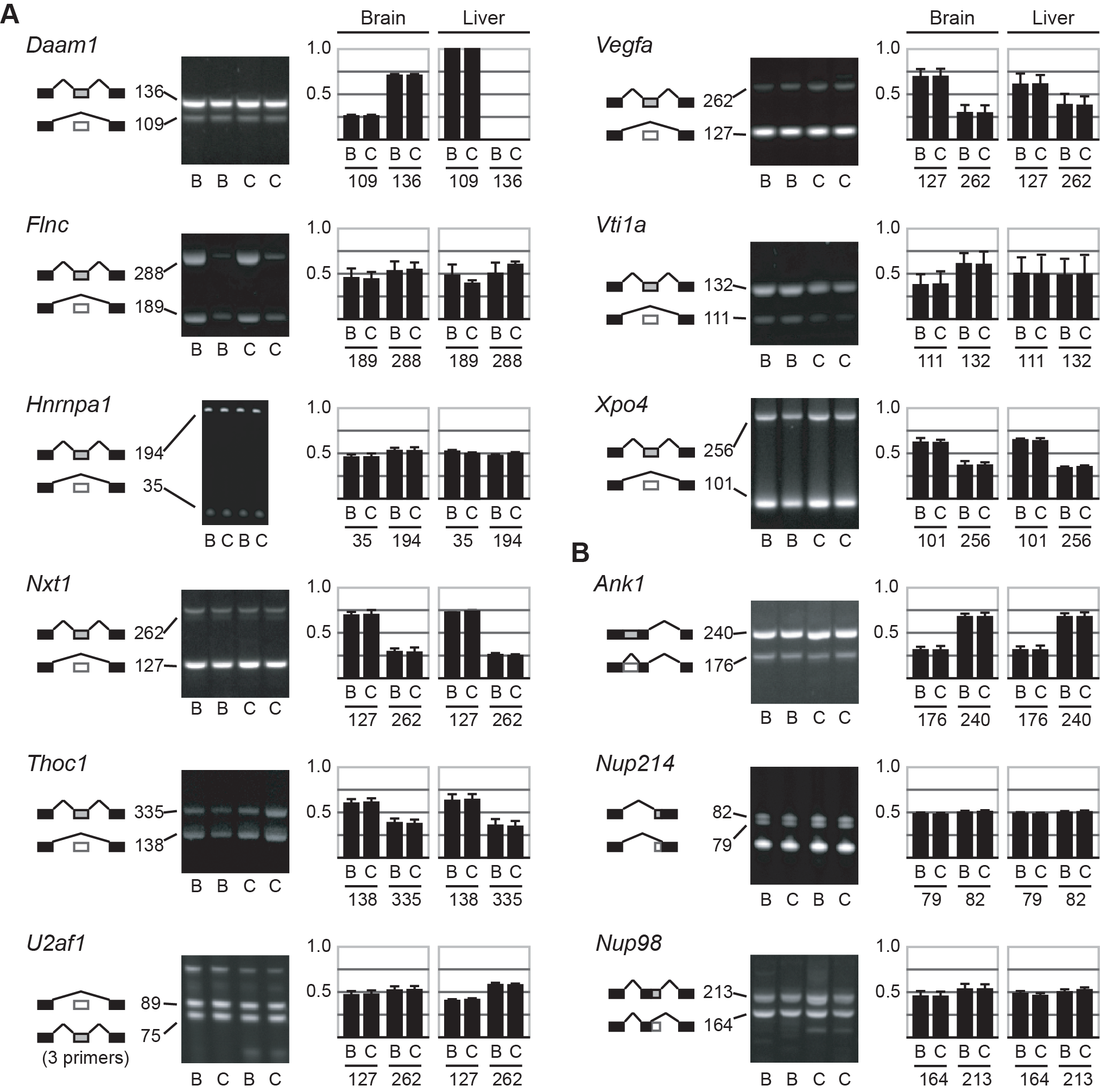

Supplement: S3 Fig — (A) Gel assays for well-characterized alternative splicing of cassette exons, including several encoding Nxf1-interacting factors, show no difference in general or tissue-specific inclusion rates between Nxf1 B6 (B) and Nxf1 CAST (C) alleles in congenic mice. Splice choice is diagrammed, with PCR product sizes indicated. Bar graphs show proportion of fluorescence intensity in each band among 13–20 independent sample pairs tested for each gene. (B) Single examples for a retained intron (Ank1), alternative splice acceptor (Nup214) and alternative splice donor (Nup98) show no difference between Nxf1 alleles. (TIF) [file pgen.1005123.s007.tif]

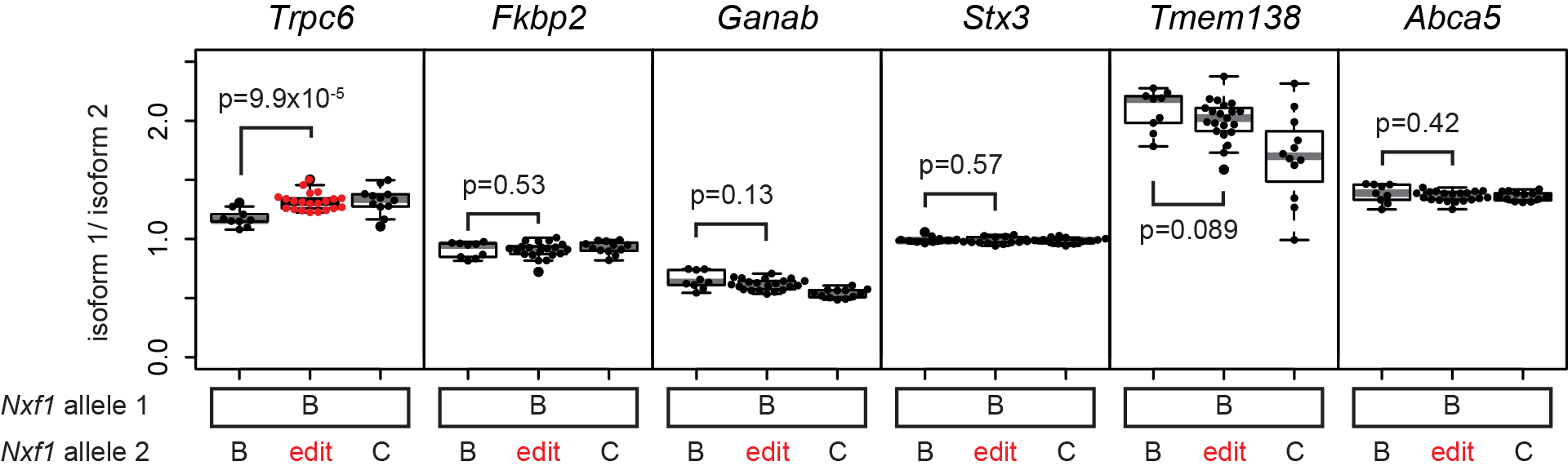

Supplement: S4 Fig — The alternative events in the congenic interval on chromosome 19 that were found by the arrays and confirmed in gel assays were re-assayed in co-isogenic E610G genome-edited animals and controls, using the samples from Fig. 6 and the assays from Fig. 3. Trpc6 gel assay, included as a positive control, showed suppression of the IAP-dependent splice form. None of the four chromosome 19 alternative events that were significant in both array (Fig. 3B) and gel (Fig. 3C) assays showed significant differences between edited and unedited heterozygotes with respect to Nxf1 B6. Indicated p-vlaues are for the one-tailed Wilcoxon rank sum test for the direction of difference seen in Fig. 3C, with the exception of Abca5, included as a negative control, which shows the value for the two-tailed test, as it had no predicted difference. Inclusion of heterozygous congenic samples confirms the effect, detection sensitivity and allele-independents effects predicted from Fig. 3C for Ganab (p = 0.00015) and Tmem138 (p = 0.0036), but not for Fkbp2 (p = 0.28) or Stx3 (p = 0.40), albeit comparing new edited with archived congenic samples. (TIF) [file pgen.1005123.s008.tif]

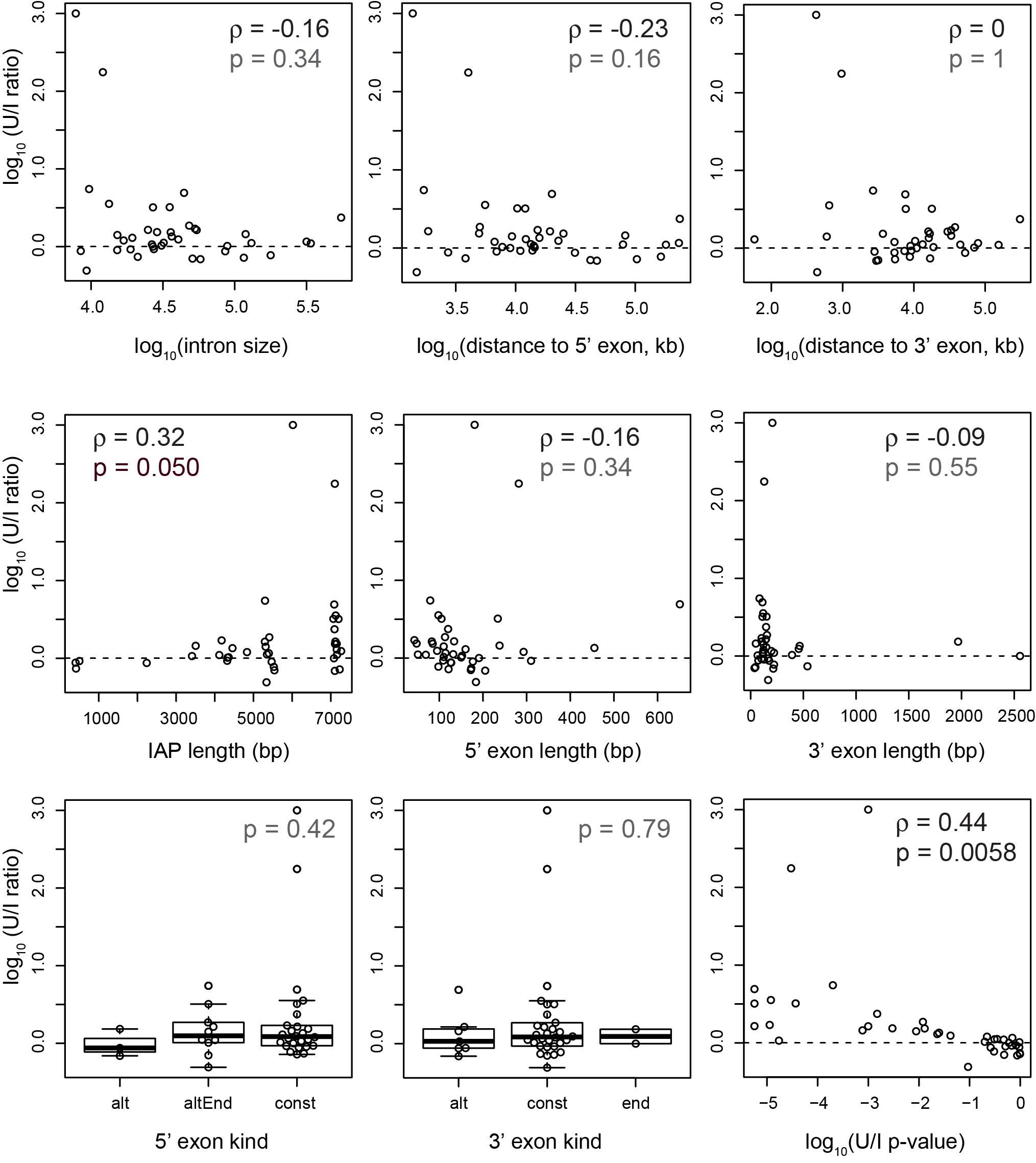

Supplement: S5 Fig — Plots show relationships between indicated attributes of the studied introns (x-axes) and expression ratio between uninserted strain and inserted strains with respect to the IAP (U/I ratio). U/I ratio is expressed as log10 to permit inclusion of exceptionally strong effects at Adamts13 and Zfp69. Dashed line at zero indicates elements with no measureable effect. Spearman correlation coefficients (ρ) and associated p-values are shown. For categorical annotations of alternatively spliced (alt), alternative 3’ end formation (altEnd), constitutive (con), and 3’ terminal (end) exons, p-values are derived from the Kruskal-Wallis test. The magnitude of U/I ratio is moderately well correlated with its p-value, as expected for events near the threshold for discrimination and in the presence of other potential modifying effects across strains. (TIF) [file pgen.1005123.s009.tif]

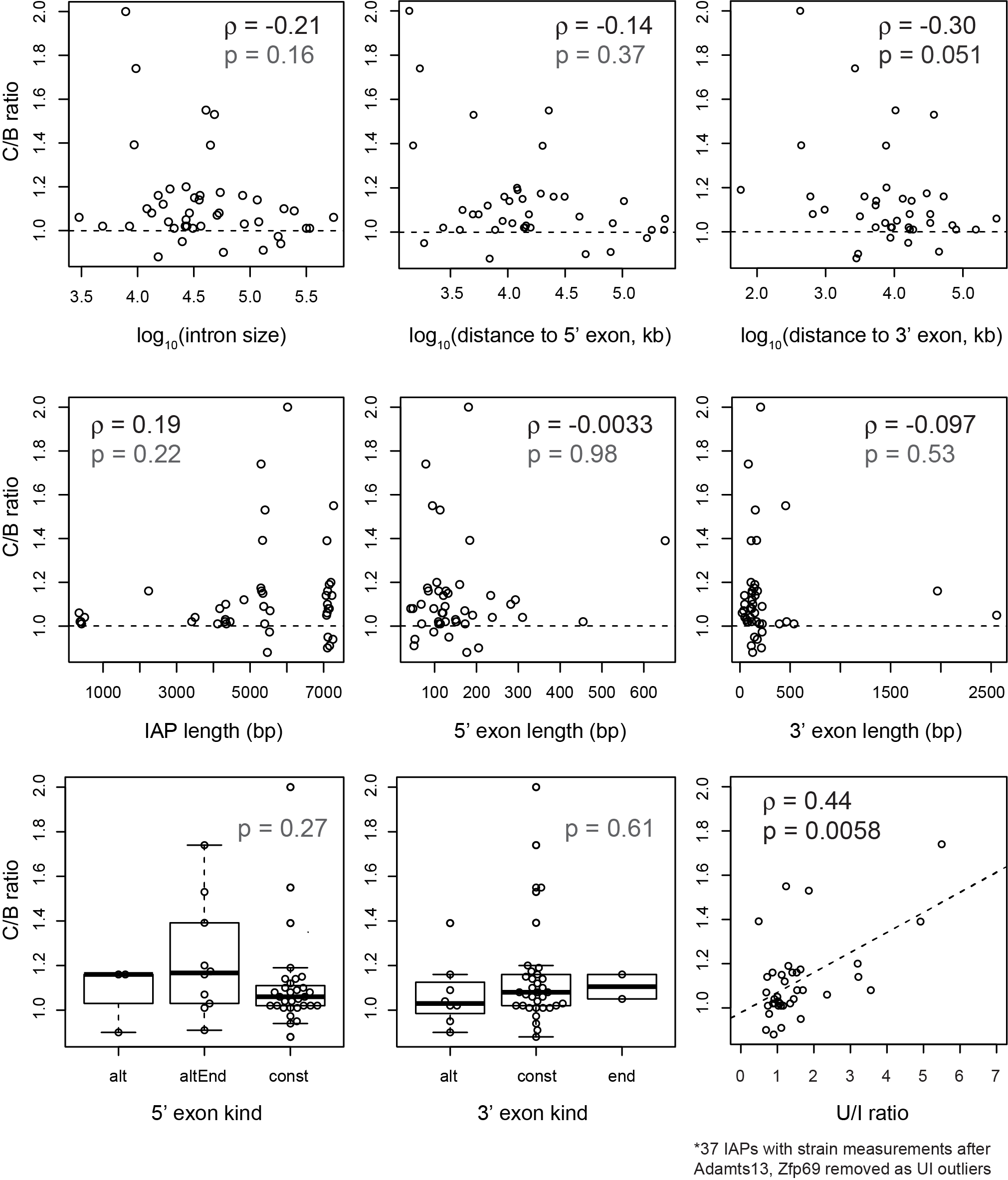

Supplement: S6 Fig — Plots show relationships between indicated attributes of the studied introns (x-axes) and expression ratio between Nxf1 CAST and Nxf1 B6 alleles (C/B ratio). For categorical annotations of alternatively spliced (alt), alternative 3’ end formation (altEnd), constitutive (con), and 3’ terminal (end) exons, p-values are derived from the Kruskal-Wallis test. Nominal evidence for correlation to distance from 3’ exon does not survive correction for multiple tests, but may merit further study. As expected, C/B ratio is correlated with U/I ratio, as larger mutational effects should provide a larger phenotypic space for suppression. (TIF) [file pgen.1005123.s010.tif]

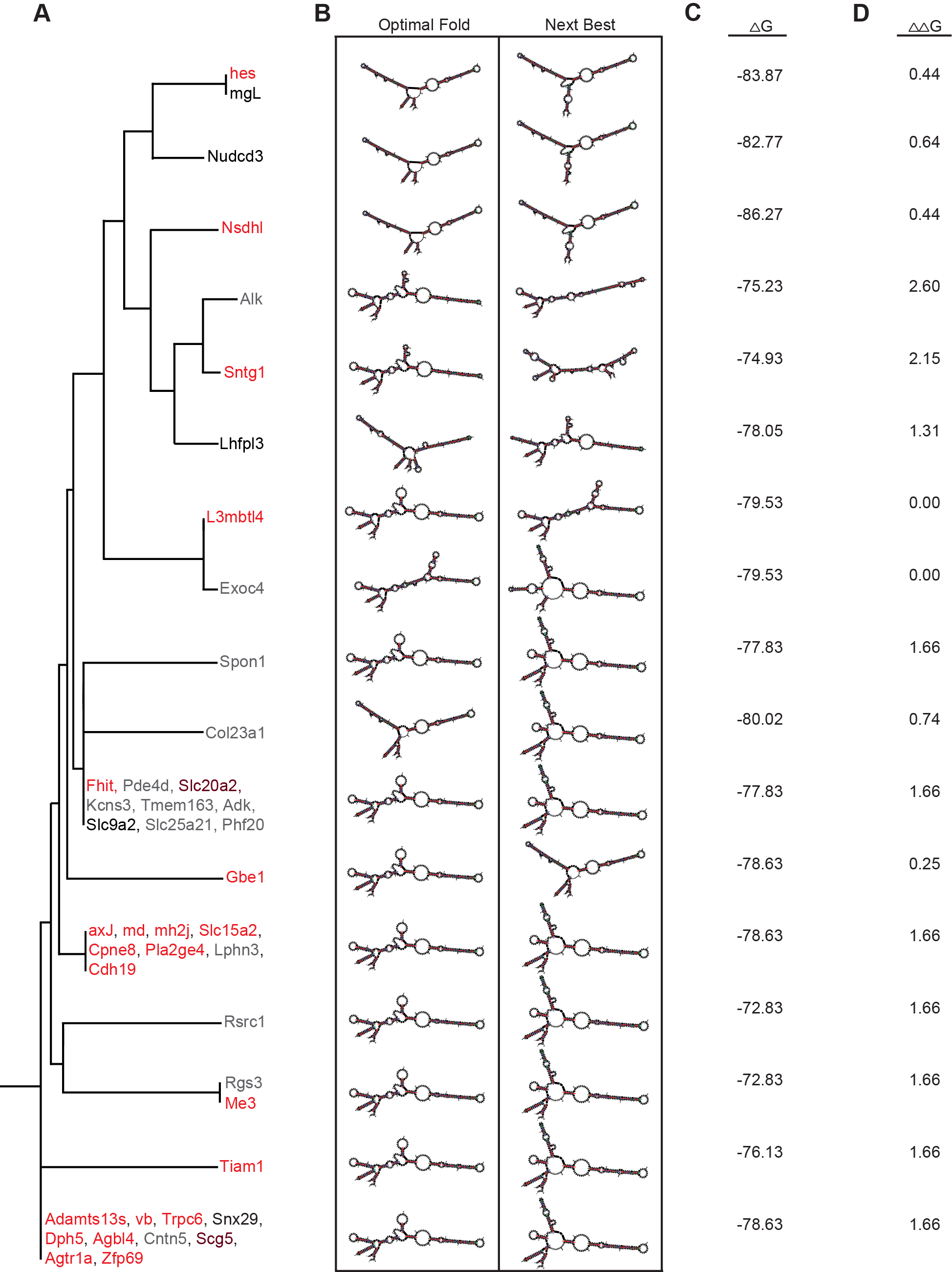

Supplement: S8 Fig — Secondary structures of RTE RNAs were predicted using the mfold web server version 3.4 (http://mfold.rna.albany.edu/?q=mfold/download-mfold) under default parameters. (A) RTE phylogeny, as in S7 Fig, panel J. (B) Optimal (left column) and next best (right column) predicted secondary structures of RTE RNA sequence for each unique phylogeny group, based on calculated lowest free energy, are depicted. (C) Free energy of optimal structures, and (D) difference in free energy between optimal and next best secondary structures are listed. In addition to identical RTE sequences with divergent outcomes, RTE structures from adjacent positions in the tree also showed dissimilar effects (e.g., hes, mgL, Nudcd3, and Nsdhl). (TIF) [file pgen.1005123.s012.tif]
