# Supplementary material for: Nxf1 Natural Variant E610G Is a Semi-dominant Suppressor of IAP-Induced RNA Processing Defects
Source: PLoS Genet. 2015 Apr 2;11(4):e1005123. doi: 10.1371/journal.pgen.1005123 (PMC4383553; doi:10.1371/journal.pgen.1005123)

A: Full length IAPs

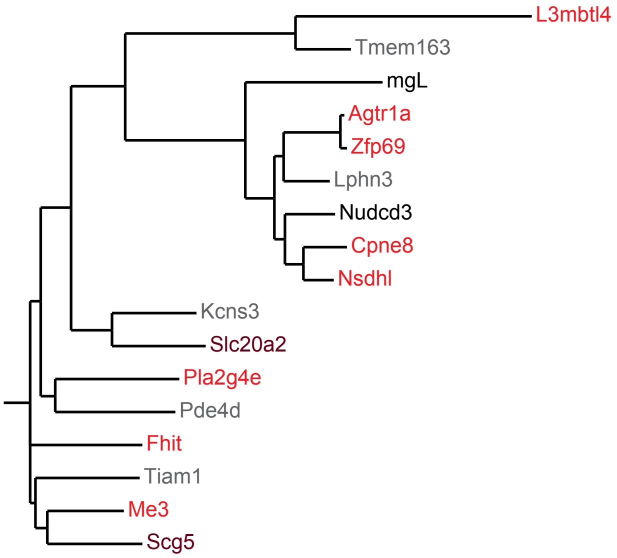

Alignment length: 7521
Input median: 7119
Input range: 7065-7268

B: IΔ1 IAPs

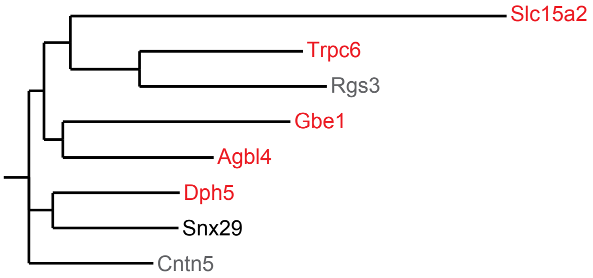

Alignment length: 5604
Input median: 5343
Input range: 5295-5471

C: LTRs

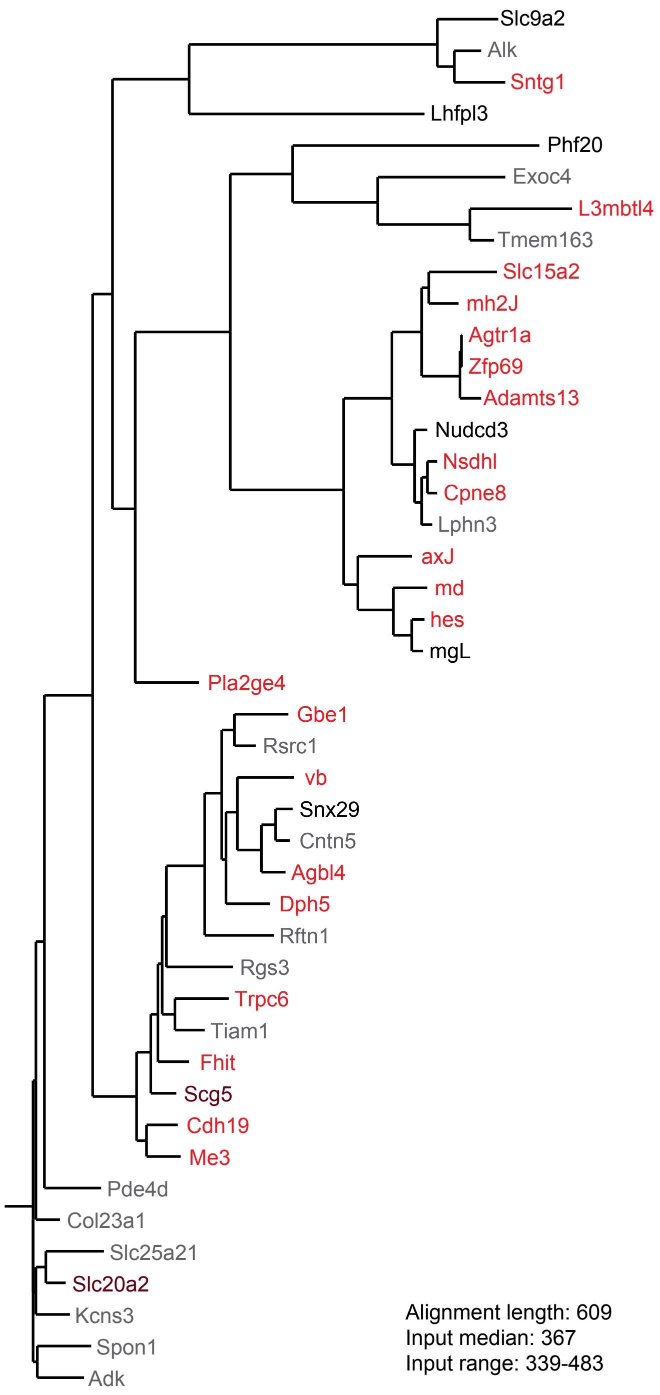

Alignment length: 609
Input median: 367
Input range: 339-483

Figure S6 – p. 2

D: LTR-gag interval

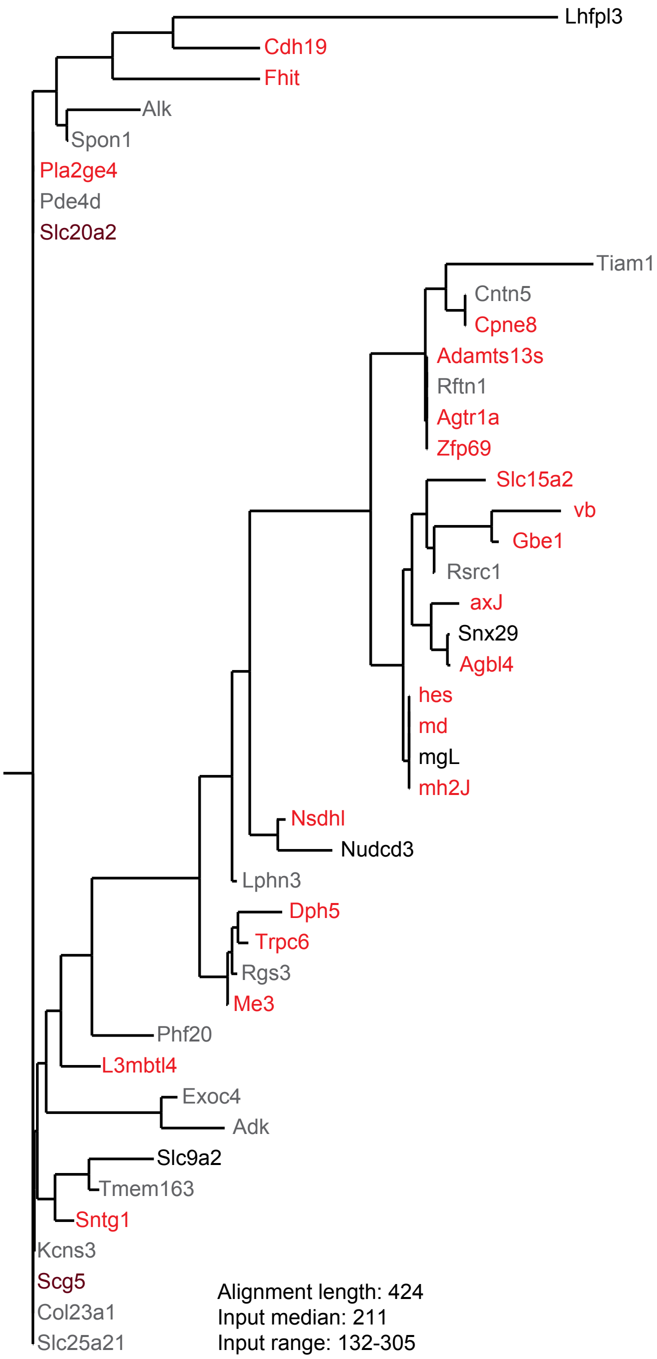

E: gag

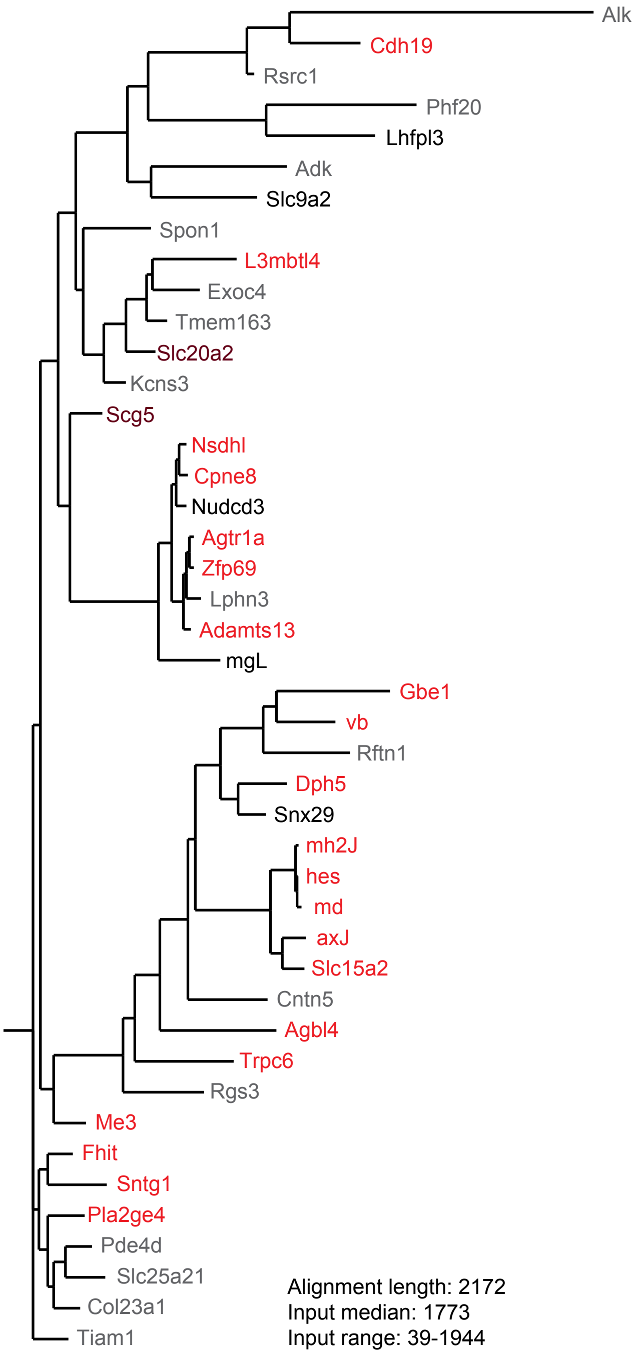

Figure S6 – p. 3

F: prt

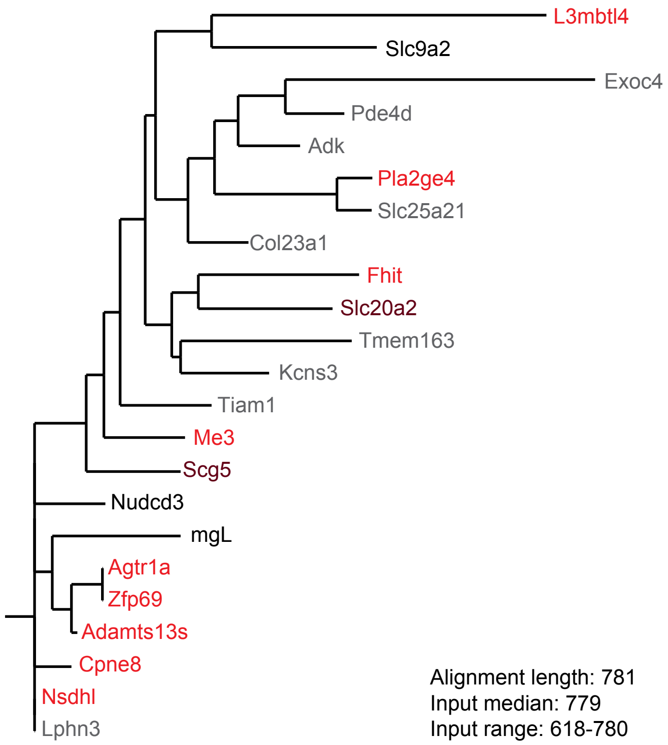

Alignment length: 781
Input median: 779
Input range: 618-780

G: prt to pol

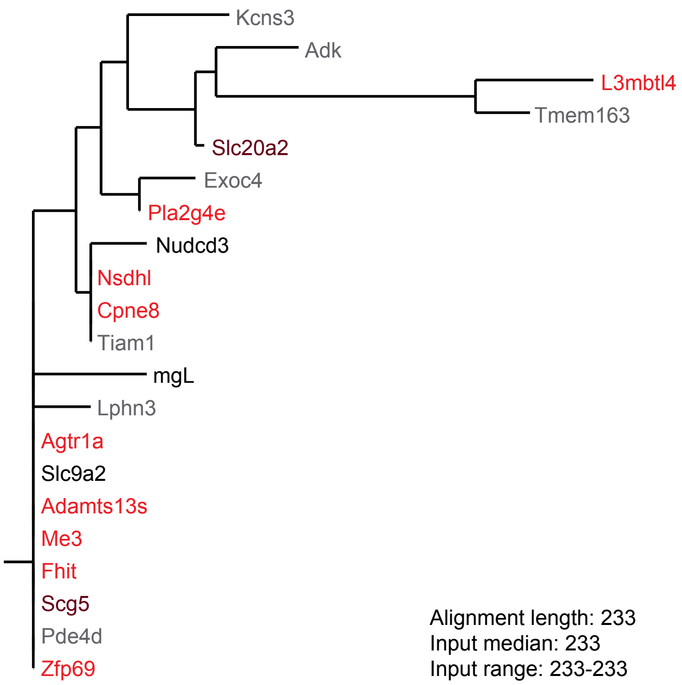

Alignment length: 233
Input median: 233
Input range: 233-233

Figure S6 - p.4

H: pol

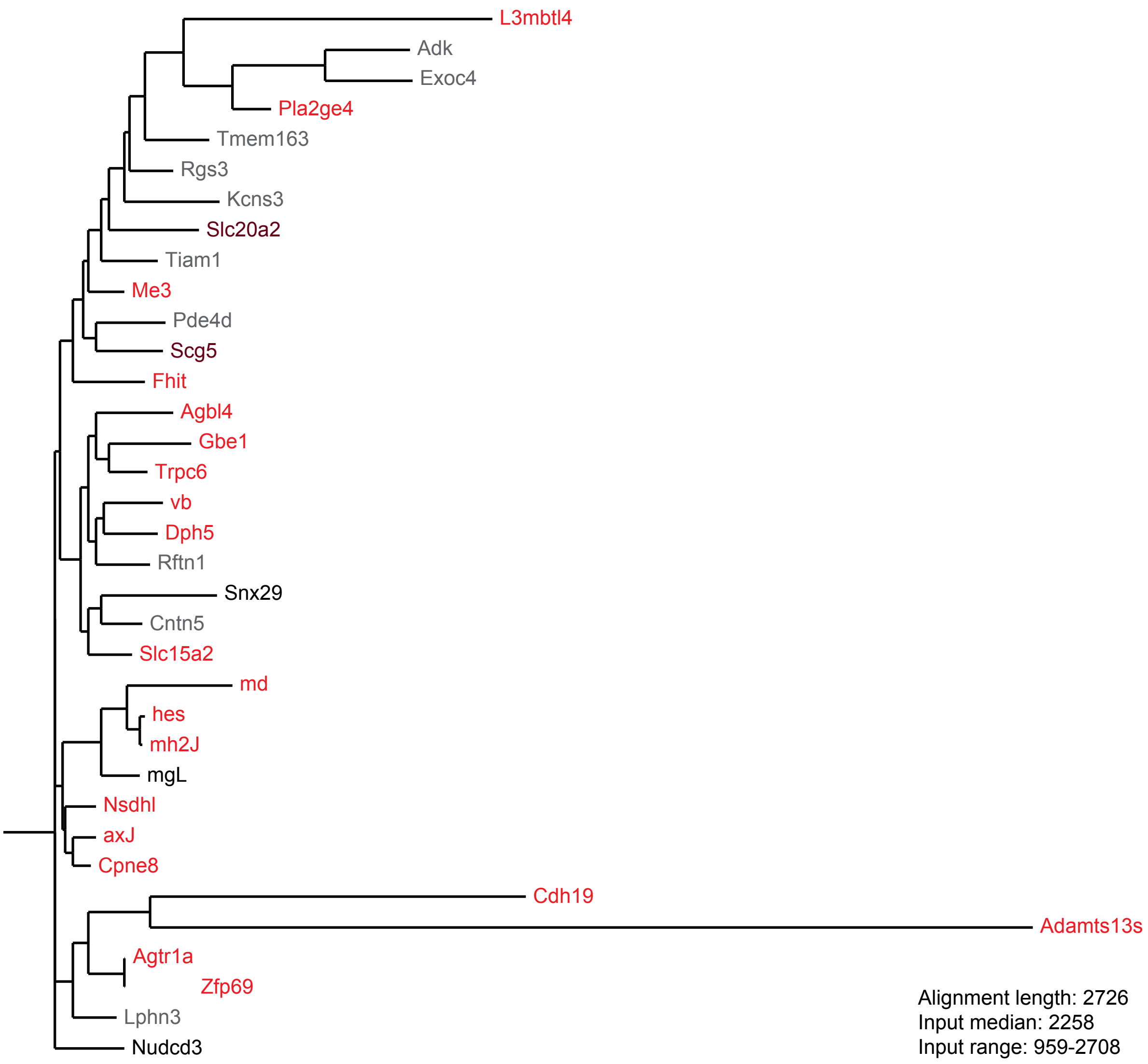

Figure S6 - p.5

I: pol to RTE

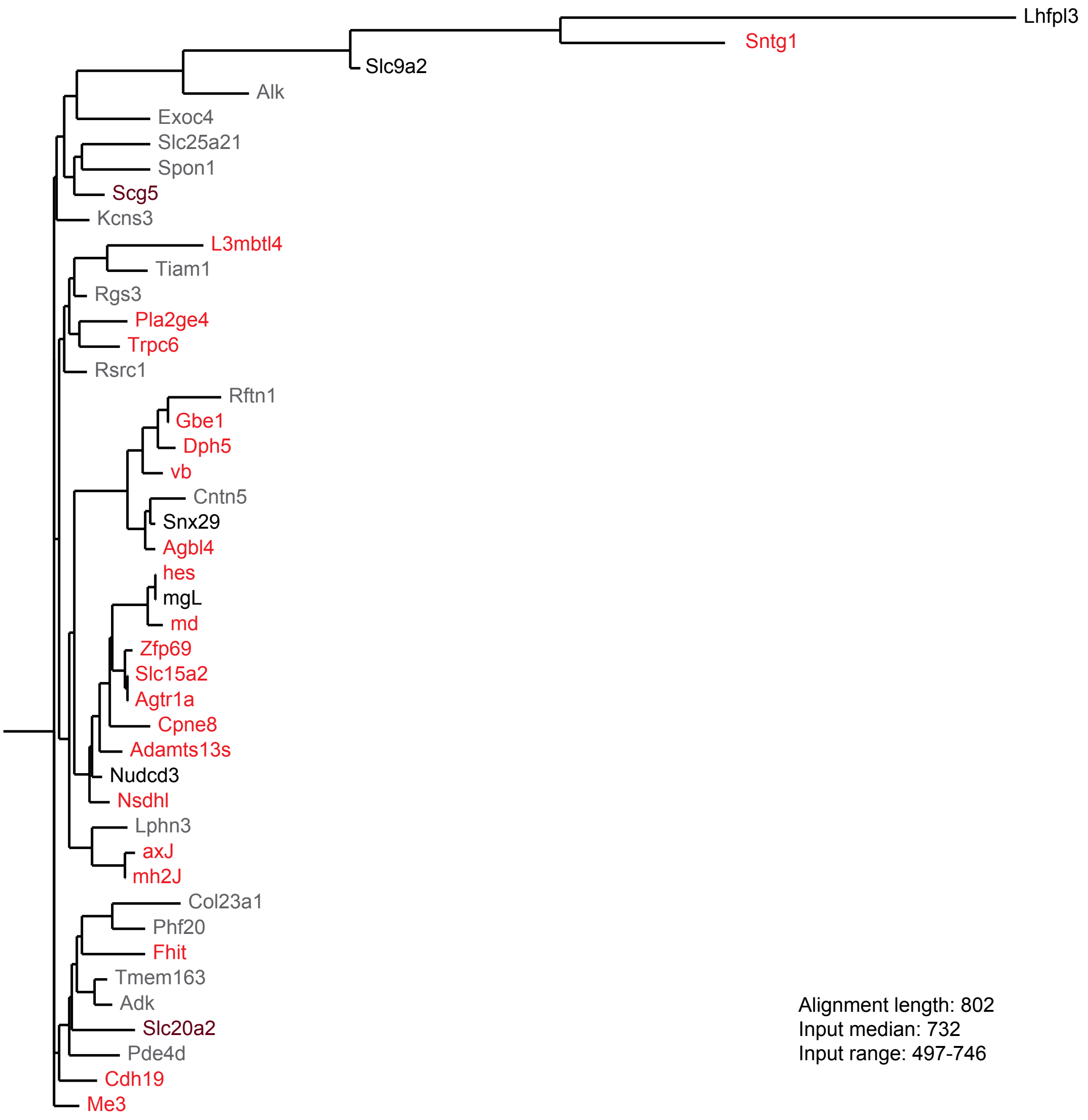

Figure S6 - p.6
J: RTE

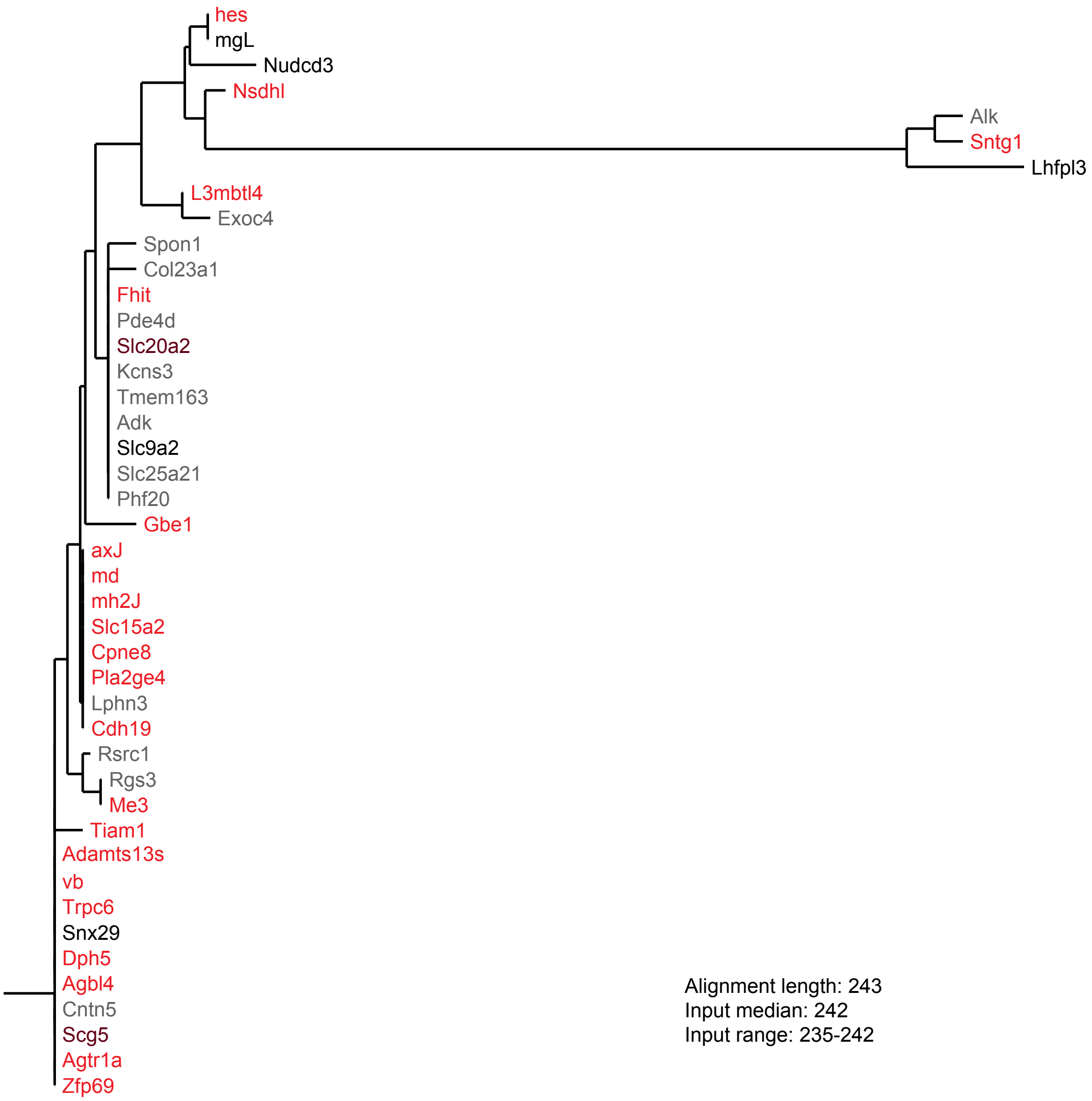

Alignment length: 243
Input median: 242
Input range: 235-242

Figure S6 - p. 7

K: ppt

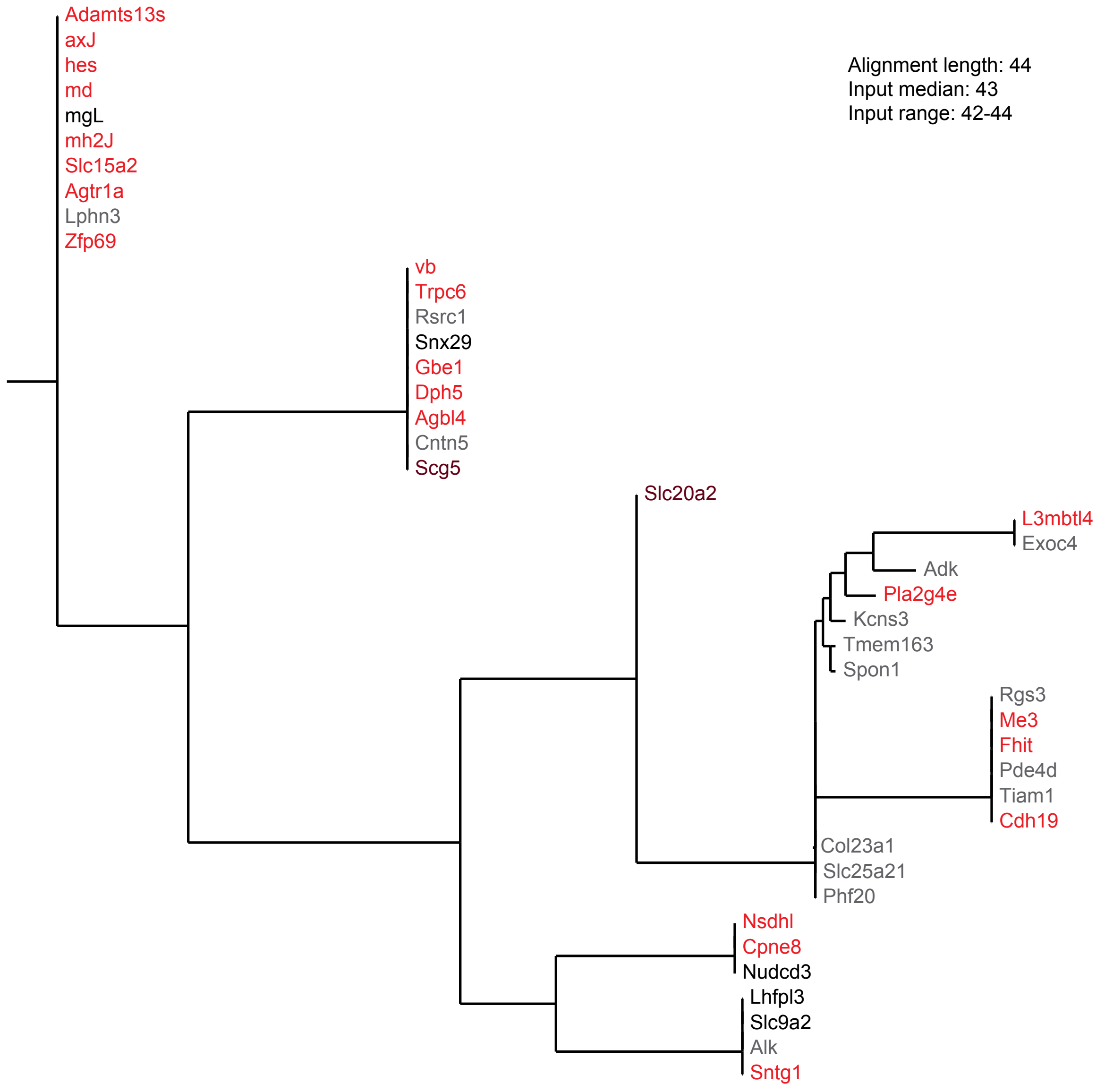

Supplement: S7 Fig — Neighbor-joining trees were generated from alignments in MUSCLE (http://www.ebi.ac.uk/Tools/msa/muscle/) for the indicated IAP groups or sequence components. Elements are colored as in Fig. 2, red for those with strong evidence for Nxf1-mediated suppression, grey for those with no evidence of mutagenic effect across strains. Nucleotide length of the total alignment, as well as lengths of the median, shortest and longest input sequences are indicated for each class or segment of IAP sequence. (A) Full alignment of IAP sequences >7kb (“full length”). (B) Full alignment of elements containing the IΔ1 deletion. Alignments of specific sequence components are (C) LTRs, (D) interval between LTR and gag, (E) gag, (F) prt, (G) prt to pol, (H) pol, (I) pol to the RTE, (J) RTE, (K) ppt. (PDF) [file pgen.1005123.s011.pdf]
